# A multidisciplinary approach and consensus statement to establish standards of care for Angelman syndrome

**DOI:** 10.1002/mgg3.1843

**Published:** 2022-02-11

**Authors:** Jessica Duis, Mark Nespeca, Jane Summers, Lynne Bird, Karen G.C.B. Bindels‐de Heus, M. J. Valstar, Marie‐Claire Y. de Wit, C. Navis, Maartje ten Hooven‐Radstaake, Bianca M. van Iperen‐Kolk, Susan Ernst, Melina Dendrinos, Terry Katz, Gloria Diaz‐Medina, Akshat Katyayan, Srishti Nangia, Ronald Thibert, Daniel Glaze, Christopher Keary, Karine Pelc, Nicole Simon, Anjali Sadhwani, Helen Heussler, Anne Wheeler, Caroline Woeber, Margaret DeRamus, Amy Thomas, Emily Kertcher, Lauren DeValk, Kristen Kalemeris, Kara Arps, Carol Baym, Nicole Harris, John P. Gorham, Brenda L. Bohnsack, Reid C. Chambers, Sarah Harris, Henry G. Chambers, Katherine Okoniewski, Elizabeth R. Jalazo, Allyson Berent, Carlos A. Bacino, Charles Williams, Anne Anderson

**Affiliations:** ^1^ Section of Genetics & Inherited Metabolic Disease Section of Pediatrics, Special Care Department of Pediatrics Children’s Hospital Colorado University of Colorado Anschutz Medical Campus Aurora Colorado USA; ^2^ Department of Neurology Rady Children’s Hospital San Diego California USA; ^3^ Department of Psychiatry The Hospital for Sick Children Toronto Ontario Canada; ^4^ Department of Pediatrics Clinical Genetics / Dysmorphology University of California, San Diego Rady Children’s Hospital San Diego San Diego California USA; ^5^ Department of Pediatrics Erasmus MC Sophia Children’s Hospital Rotterdam Netherlands; ^6^ ENCORE Expertise Center for Neurodevelopmental Disorders Erasmus MC University Medical Center Rotterdam The Netherlands; ^7^ Department of Neurology and Pediatric Neurology Erasmus MC Rotterdam The Netherlands; ^8^ Department of ENT (Speech & Language Pathology) Erasmus MC Rotterdam The Netherlands; ^9^ Department of Physical Therapy Erasmus MC Rotterdam The Netherlands; ^10^ Department of Obstetrics and Gynecology University of Michigan Ann Arbor Michigan USA; ^11^ Developmental Pediatrics Department of Pediatrics Children’s Hospital Colorado University of Colorado Anschutz Medical Campus Aurora CO USA; ^12^ Division of Neurology and Developmental Pediatrics Department of Pediatrics Baylor College of Medicine Houston Texas USA; ^13^ Neurology Texas Children's Hospital Houston Texas USA; ^14^ Department of Pediatrics Division of Child Neurology Weill Cornell Medicine New York‐Presbyterian Hospital New York New York USA; ^15^ Angelman Syndrome Program Lurie Center for Autism Massachusetts General Hospital for Children Boston Massachusetts USA; ^16^ Department of Neurology Hôpital Universitaire des Enfants Reine Fabiola Université Libre de Bruxelles (ULB) Brussels Belgium; ^17^ Department of Psychiatry Boston Children’s Hospital Boston MA USA; ^18^ UQ Child Health Research Centre Faculty of Medicine The University of Queensland Brisbane Queensland Australia; ^19^ Center for Newborn Screening RTI International Research Triangle Park North Carolina USA; ^20^ Audiology, Speech & Learning Services Children’s Hospital Colorado Aurora Colorado USA; ^21^ Department of Psychiatry Carolina Institute for Developmental Disabilities University of North Carolina at Chapel Hill Chapel Hill North Carolina USA; ^22^ New York League for Early Learning William O'connor School New York New York USA; ^23^ UNC Hospital Chapel Hill Chapel Hill North Carolina USA; ^24^ Occupational Therapy Children’s Hospital Colorado Aurora Colorado USA; ^25^ Department of Pediatric Rehabilitation Monroe Carell Jr. Children's Hospital at Vanderbilt Nashville Tennessee USA; ^26^ Department of Physical Therapy Children’s Hospital Colorado University of Colorado Anschutz Medical Campus Aurora Colorado USA; ^27^ Physical Therapy Children’s Hospital Colorado Aurora Colorado USA; ^28^ Department of Ophthalmology and Visual Sciences University of Michigan Ann Arbo Michigan USA; ^29^ Division of Ophthalmology Department of Ophthalmology Ann & Robert H. Lurie Children’s Hospital of Chicago Northwestern University Feinberg School of Medicine Ann Arbo Michigan USA; ^30^ Department of Orthopedic Surgery Nationwide Children’s Hospital Columbus Ohio USA; ^31^ Orthopedic Surgery San Diego Department of Pediatric Orthopedics University of California Rady Children’s Hospital San Diego California USA; ^32^ Angelman Syndrome Foundation Aurora Illinois USA; ^33^ Foundation for Angelman Syndrome Therapeutics Chicago Illinois USA; ^34^ Department of Molecular and Human Genetics Baylor College of Medicine Houston Texas USA; ^35^ Raymond C. Philips Unit Division of Genetics and Metabolism Department of Pediatrics University of Florida Gainesville Florida USA

**Keywords:** Angelman Syndrome, genetics, management, neurogenetics, UBE3A

## Abstract

**Background:**

Angelman syndrome (AS) is a rare neurogenetic disorder present in approximately 1/12,000 individuals and characterized by developmental delay, cognitive impairment, motor dysfunction, seizures, gastrointestinal concerns, and abnormal electroencephalographic background. AS is caused by absent expression of the paternally imprinted gene *UBE3A* in the central nervous system. Disparities in the management of AS are a major problem in preparing for precision therapies and occur even in patients with access to experts and recognized clinics. AS patients receive care based on collective provider experience due to limited evidence‐based literature. We present a consensus statement and comprehensive literature review that proposes a standard of care practices for the management of AS at a critical time when therapeutics to alter the natural history of the disease are on the horizon.

**Methods:**

We compiled the key recognized clinical features of AS based on consensus from a team of specialists managing patients with AS. Working groups were established to address each focus area with committees comprised of providers who manage >5 individuals. Committees developed management guidelines for their area of expertise. These were compiled into a final document to provide a framework for standardizing management. Evidence from the medical literature was also comprehensively reviewed.

**Results:**

Areas covered by working groups in the consensus document include genetics, developmental medicine, psychology, general health concerns, neurology (including movement disorders), sleep, psychiatry, orthopedics, ophthalmology, communication, early intervention and therapies, and caregiver health. Working groups created frameworks, including flowcharts and tables, to help with quick access for providers. Data from the literature were incorporated to ensure providers had review of experiential versus evidence‐based care guidelines.

**Conclusion:**

Standards of care in the management of AS are keys to ensure optimal care at a critical time when new disease‐modifying therapies are emerging. This document is a framework for providers of all familiarity levels.

## 1 INTRODUCTION

Angelman syndrome (AS, MIM# 105830) is a neurogenetic disorder impacting approximately 1/12,000 to 1/20000 (Steffenburg et al., 1996). Diagnosis of classic AS is often made between 1 and 2 years of age, often later with non‐classical presentation (Gentile et al., 2010). Characteristic features include developmental delays especially in expressive language, a distinctive happy demeanor, seizures, gross and fine motor deficits, tremors, sleep disturbances, gastrointestinal problems, stereotypical behavior, anxiety, and hyperkinesis.

**FIGURE 1:**
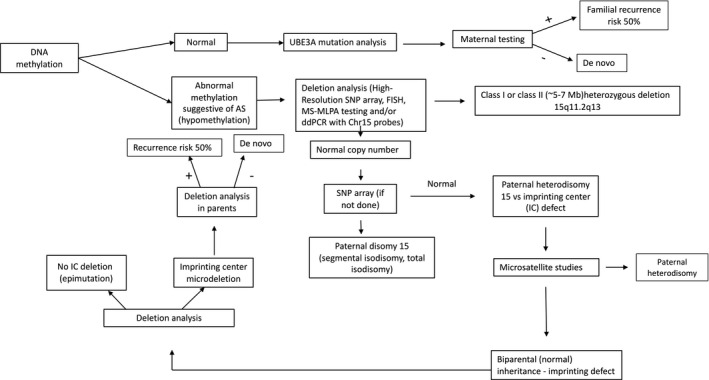
Angelman Syndrome (AS) Genetic Testing Algorithm. Guidance to work up when considering a diagnosis of AS. Abbreviations: FISH Fluorescence in situ hybridization; SNP single nucleotide polymorphism; MS methylation‐specific; MLPA multiplex ligation‐dependent probe amplification; PWS Prader‐Willi Syndrome; AS Angelman syndrome; UPD uniparental disomy; *UBE3A* ubiquitin protein ligase E3A gene.

The ubiquitin protein E3A ligase gene (*UBE3A*) is paternally imprinted in neurons, and the clinical features of AS are primarily due to deficient maternally expressed UBE3A protein in the brain. *UBE3A* is located on chromosome 15q11.2q13 and encodes three EA6P protein isoforms via differential splicing (Copping et al., 2017; Dindot et al., 2008; Hillman et al., 2017; Yamamoto et al., 1997). There are four recognized molecular mechanisms: (a) deletion of maternal 15q11.2q13 (70–75%); (b) mutation of maternal *UBE3A* gene (15%) with a 50% recurrence risk if maternally inherited; rarely (c) paternal uniparental disomy (UPD, 5–7%); and (d) imprinting defect (ImpD, 5–7%) due to an epimutation conferring an aberrant paternal imprint onto the maternal *UBE3A*, or less commonly due to deletion of the imprinting center with a 50% risk of recurrence if maternally inherited. Mosaicism is primarily associated with an epimutation (Aypar et al., 2016; Camprubi et al., 2007; Carson et al., 2019; Fairbrother et al., 2015). Features vary depending upon molecular subtype (Bindels‐de Heus et al., 2020; Larson et al., 2015; Sahoo et al., 2007; Tan et al., 2011). Figure 1 presents a diagnostic algorithm. Genetic counseling should be offered to all families to discuss recurrence risk, which differs based on molecular subtype.

This recommendation aims to highlight standardization of care in preparation for personalized therapies. The Food and Drug Administration recommends establishing an accessible document with an expert statement regarding treatment.

## 2 METHODS

The consensus of care across the lifespan reflects the compilation of input from more than 20 key opinion leaders (KOLs) in the field. The document was circulated to experts across the globe for feedback and revision. All co‐authors met at least one of the following criteria: (a) substantial clinical experience working with children and adults with AS (operationalized as having seen at least 5 individuals in both consultation and follow‐up) and (b) willingness to practice evidence‐based medicine with comprehensive review of the literature.

Literature review was performed in PubMed and Ovid Medline. PubMed search terms included (Angelman Syndrome) AND (Management OR treatment OR clinical trial OR medication OR behavioral OR intervention), yielding 1,273 articles. The Ovid search terms included Angelman Syndrome/dg, dh, dt, pc, th [Diagnostic Imaging, Diet Therapy, Drug Therapy, Prevention & Control, Therapy] AND (Management OR treatment OR clinical trial OR medication OR behavioral OR intervention).mp. [mp=title, abstract, original title, name of substance word, subject heading word, floating sub‐heading word, keyword heading word, organism supplementary concept word, protocol supplementary concept word, rare disease supplementary concept word, unique identifier, synonyms] and yielded 59 results. Articles included were clinical trials, retrospective case reviews, meeting abstracts, and case series focused on the management of AS. The final review included removal of duplicates and sorting for relevance to human management and yielded 100 articles.

## 3 REVIEW AND PRACTICE GUIDELINES

Through data gathered in natural history studies and clinical experience, the clinical description continues to evolve to include newly recognized features. Due to the limited nature of relevant references and a lack of evidence base, KOL experience was prioritized over the literature review, but both were considered. A limited number of clinical trials to support practices were available, and therefore, the evidence level is III (controlled trials without randomization) or IV (designed case‐control or cohort studies).

### 3.1 General considerations

Table 1 shows comprehensive management by age.

**TABLE 1:** Health considerations by age for individuals with Angelman syndrome

Age	Medical eval	Anticipatory guidance	Medical referrals	Labs	Diagnostic	Medication/supplement considerations
Age of diagnosis	Feeding Vision ‐ CVI ‐ strabismus GERD Growth & Development Tone Seizures	Genetic counseling Hold upright during feeding and for 30 minutes after feeding (GERD precautions) Discuss low‐carbohydrate, higher protein and fat foods towards implementation of LGIT diet Early intervention services Assess sleep Seizures precautions/management Monitor constipation Sufficient environmental stimulation Support groups Clinical research^a^	Genetics/Genetic Counseling Neurology Ophthalmology GI/Nutrition OT ‐ Address sensory needs PT ‐ Orthotics ‐ Aqua therapy SLP ‐ AAC	Genetic confirmation (See Figure 1) If failure to thrive is present: CMP, CBC, thyroid studies, vitamin D, magnesium, phosphorus Assess patient produces ketones as expected if initiating diet: acylcarnitine profile, urine organic acids, free and total carnitine Additional labs before initiating/monitoring diet: selenium, zinc, ionized calcium, BHB, lipid panel, carnitine, urine calcium Ferritin with ESR	Hip x‐ray (especially if not ambulatory) Spine x‐ray EEG, especially if suspect seizures Feeding evaluation ‐ VFSS if needed	Diet: LGIT or ketogenic diet Seizure management (see Figure 2) MCT oil to support diet/constipation Levocarnitine if level borderline or low in patient on low carbohydrate diet
1–3 years old	Growth & development Vision ‐ CVI ‐ strabismus Feeding Seizures Sleep Behavior	Early intervention services LGIT/ketogenic diets Routines ‐ Bedtime ‐ Toileting (see Figure S1) ‐ Daily activities ‐ Behavioral modification Limit‐setting Seizures Support groups Clinical research^a^	Neurology Medical Home/AS specialist Ophthalmology Developmental Pediatrician GI/Nutrition Sleep (if not addressed by another specialist) OT ‐ Address sensory needs PT ‐ Orthotics ‐ Aqua therapy ‐ hippotherapy SLP ‐ AAC Vision therapy Equipment referral (specialized stroller, car seat, Safe sleep bed) Applied behavioral analysis/behavioral therapy Dental care	Ferritin and ESR CBC Vitamin D Diet Monitoring ‐ CBC ‐ Vitamin D ‐ CMP ‐ Selenium ‐ Magnesium ‐ Phosphorus ‐ Zinc ‐ Carnitine ‐ BHB ‐ Lipid panel ‐ Urine calcium ‐ Ionized calcium	Hip x‐ray (especially if not ambulatory) Spine x‐ray Consider EEG Feeding evaluation	Diet: LGIT or ketogenic diet Seizure management (see Figure 2) MCT oil to support diet/constipation Levocarnitine if level borderline or low in patient on low carbohydrate diet Sleep Management (see Figure 3) Consider transition to Safe Sleep bed Behavior Management (see Figure 4) Treat constipation with stool softener +mild stimulant (e.g. senna, magnesium)
1–5 years old	Growth & development Seizures Vision ‐ CVI ‐ strabismus Feeding Scoliosis Sleep Behavior Mobility	Early intervention services/IEP preparation Seizures LGIT/ketogenic diets Routines ‐ Bedtime ‐ Toileting (see Figure S1) ‐ Daily activities ‐ Behavioral modification strategy Limit‐setting Constipation (can be linked to sleep disturbance, seizures, behavior changes) Activity ‐ Adaptive sports ‐ Exercise 30–90 minutes per day Monitor gait over time Sleep ‐ Consider role of seizures at night Support groups Clinical research^a^	Neurology Medical Home/AS specialist Developmental Pediatrician (if not addressed by another specialist) Sleep (if not addressed by another specialist) Ophthalmology GI/Nutrition SLP ‐ AAC focus OT PT ‐ Orthotics ‐ strengthening ‐ Aqua therapy ‐ Hippotherapy ‐ SPIDER therapy Vision therapy Applied behavioral analysis/behavioral therapy Dental care	Ferritin and ESR Vitamin D Diet Monitoring ‐ CBC ‐ Vitamin D ‐ CMP ‐ Selenium ‐ Magnesium ‐ Phosphorus ‐ Zinc ‐ Carnitine ‐ BHB ‐ Lipid panel ‐ Urine calcium ‐ Ionized calcium	Hip x‐ray (especially if not ambulatory) Spine x‐ray Consider EEG Feeding evaluation Consider sleep study (best if in home environment) DEXA scan every 2 years if on low carbohydrate diet	Diet: LGIT or ketogenic diet Seizure management (see Figure 2) MCT oil to support diet/constipation Levocarnitine if level borderline or low in patient on low carbohydrate diet Sleep Management (see Figure 3) Consider transition to Safe Sleep bed Behavior Management (see Figure 4) Treat constipation daily with stool softener +mild stimulant (e.g. senna)
5–13 years‐old	Growth & development Seizures Sleep Behavior Vision Scoliosis Mobility Weight management	Seizures Non‐epileptic myoclonus may emerge around the time of puberty Sleep LGIT/ketogenic diets Hyperphagia Constipation Mobility (change in gait pattern, consider pain) Constipation (can be linked to sleep disturbance, seizures, behavior changes) Anxiety Puberty ‐ Monitor seizures ‐ Behavior changes ‐ Plan for suppression of menses (in females) Routines/consistency in all environments ‐ Bedtime ‐ Toileting (see Figure S1) ‐ Daily activities ‐ Behavioral modification strategy ‐ Safety plan (tracking if elopement is a concern) IEP intervention ‐ PT^b^ ‐ SLP: AAC integration ‐ OT: focus on independence, activities of daily living ‐ Para pro ‐ Inclusion where appropriate ‐ Functional behavioral assessment and ABA/behavioral therapy services ‐ Seizure plan (prophylactic medications) • Clinical Research^a^	Neurology Medical Home/AS specialist Ophthalmology GI/Nutrition Sleep (if not addressed by another specialist) Orthopedics (as needed for mobility, scoliosis, DDH) Obstetrics & gynecology SLP ‐ AAC focus OT PT ‐ Orthotics ‐ strengthening ‐ Aqua therapy ‐ Hippotherapy ‐ SPIDER therapy Vision therapy Applied behavioral analysis/behavioral therapy Dental care IEP advocate	Ferritin and ESR Vitamin D CMP CBC Lipid panel Diet Monitoring ‐ CBC ‐ Vitamin D ‐ CMP ‐ Selenium ‐ Magnesium ‐ Phosphorus ‐ Zinc ‐ Carnitine ‐ BHB ‐ Lipid panel ‐ Urine calcium ‐ Ionized calcium	Hip x‐ray (especially if not ambulatory) Spine x‐ray NEM: rule out underlying causes – constipation, worsening sleep, decreased appetite and poor nutrition, changes in mobility related to decreased ROM and pain) DEXA every 2 years if on low carbohydrate diet long‐term, non‐ambulatory, delayed puberty or history of >2 fractures	Diet: LGIT or ketogenic diet Seizure management (see Figure 2) MCT oil to support diet/constipation Levocarnitine if level borderline or low in patient on low carbohydrate diet Sleep Management (see Figure 3) Consider transition to Safe Sleep bed Behavior Management (see Figure 4) Treat constipation daily with stool softener +mild stimulant (e.g. senna)
13–21 years‐old	Independence with ADLs Transition Seizures Sleep Behavior Scoliosis Mobility AAC use and integration Weight management	Seizures Non‐epileptic myoclonus may emerge around the time of puberty Sleep LGIT/ketogenic diets Hyperphagia Constipation Mobility (change in gait pattern, consider pain) Constipation (can be linked to sleep disturbance, seizures, behavior changes) Anxiety Puberty ‐ Monitor seizures ‐ Behavior changes ‐ Plan for suppression of menses (in females) Routines/consistency in all environments Bedtime Toileting (see Figure S1) Daily activities Behavioral modification strategy Safety plan (tracking if elopement is a concern) IEP intervention PT^b^ SLP: AAC integration OT: focus on independence, ADL Para pro Inclusion where appropriate Functional behavioral assessment and behavioral therapy services Seizure plan (prophylactic medications) Clinical Trials Socialization Vocational opportunities Guardianship Transition of care Support groups DDA services Clinical Research^a^	Neurology Medical Home/AS specialist Ophthalmology Sleep (if not addressed by other specialist) GI/Nutrition Orthopedics (as needed for mobility, scoliosis) Obstetrics & gynecology SLP ‐ AAC focus OT ‐ Focus on function and ADLs PT ‐ Orthotics ‐ strengthening ‐ Aqua therapy ‐ Hippotherapy ‐ SPIDER therapy Applied behavioral analysis/behavioral therapy Dental care IEP advocate	Ferritin and ESR Vitamin D Diet Monitoring ‐ CBC ‐ Vitamin D ‐ CMP ‐ Selenium ‐ Magnesium ‐ Phosphorus ‐ Zinc ‐ Carnitine ‐ BHB ‐ Lipid panel ‐ Urine calcium Ionized calcium	Spine x‐ray NEM: rule out underlying causes – constipation, worsening sleep, decreased appetite and poor nutrition, changes in mobility related to decreased ROM and pain) DEXA every 2 years if on low carbohydrate diet long‐term, non‐ambulatory, delayed puberty or history of >2 fractures	Diet: LGIT or ketogenic diet Seizure management (see Figure 2) MCT oil to support diet/constipation Levocarnitine if level borderline or low in patient on low carbohydrate diet Sleep Management (see Figure 3) Consider transition to Safe Sleep bed Behavior Management (see Figure 4) Treat constipation daily with stool softener +mild stimulant (e.g. senna)
Adults	Independence with ADLs Seizures Sleep Behavior Scoliosis Mobility AAC use and integration Weight management	Non‐epileptic myoclonus Sleep LGIT/ketogenic diets Hyperphagia Constipation Mobility (change in gait pattern, consider pain) Introduction and/or use of AAC Constipation (can be linked to sleep disturbance, seizures, behavior changes) Anxiety Puberty ‐ Monitor seizures ‐ Behavior changes ‐ Plan for suppression of menses (in females) Routines/consistency in all environments ‐ Bedtime ‐ Toileting (see Figure S1) ‐ Daily activities ‐ Behavioral modification strategy ‐ Safety plan (tracking if elopement is a concern) Seizures Socialization Vocational training Guardianship Transition of care Support groups DDA Preventive Medicine for adults ‐ Breast examination/ mammograms ‐ Prostate examination ‐ Colonoscopy Clinical research^a^	Medical Home/AS specialist Neurology Ophthalmology Sleep (if not addressed by other specialist) GI/Nutrition Orthopedics (as needed for mobility, scoliosis) Obstetrics & gynecology SLP ‐ AAC focus OT ‐ Focus on function and ADLs PT ‐ Orthotics ‐ strengthening ‐ Aqua therapy ‐ Hippotherapy ‐ SPIDER therapy Applied behavioral analysis/behavioral therapy Dental care	Ferritin and ESR Vitamin D Diet Monitoring ‐ CBC ‐ Vitamin D ‐ CMP ‐ Selenium ‐ Magnesium ‐ Phosphorus ‐ Zinc ‐ Carnitine ‐ BHB ‐ Lipid panel ‐ Urine calcium ‐ Ionized calcium	Spine x‐ray Consider EEG Consider sleep study (best if in home environment) NEM: rule out underlying causes – constipation, worsening sleep, decreased appetite and poor nutrition, changes in mobility related to decreased ROM and pain) DEXA every 2 years if on low carbohydrate diet long‐term, non‐ambulatory, delayed puberty or history of >2 fractures DEXA scan for females >65 years old to screen for osteoporosis	Diet: LGIT or ketogenic diet Seizure management (see Figure 2) MCT oil to support diet/constipation Levocarnitine if level borderline or low in patient on low carbohydrate diet Sleep Management (see Figure 3) Consider transition to Safe Sleep bed Behavior Management (see Figure 4) Treat constipation daily with stool softener +mild stimulant (e.g. senna) Preventive healthcare as guidelines recommend for adults (e.g. Pap smear, prostate screening, breast exam, mammogram, colonoscopy); however, anesthesia is required

Abbreviations: AAC, augmentative and assistive communication device; ABA, applied behavioral analysis; ADL, activity of daily living; AS, Angelman Syndrome; BHB, beta‐hydroxybutyrate; CBC, complete blood counts; CMP, comprehensive metabolic panel; CVI, cortical visual impairment; DDA, Developmental Disabilities Administration; DEXA, dual‐energy x‐ray absorptiometry; ESR, erythrocyte sedimentation rate; GERD, gastroesophageal reflux disease; GI, gastroenterology; IEP, individualized education plan; LGIT, low glycemic index therapy; MCT, medium chain triglycerides; NEM, non‐epileptic myoclonus; OT, occupational therapy; PT, physical therapy; SLP, speech & language pathology; SPIDER, Strengthening Program of Intensive Developmental Exercises and Activities for Reaching Maximal Potential; VFSS, video fluoroscopic swallow study.

^a^ Clinical research opportunities include participation in the Global Angelman Registry, Natural History Study, and currently recruiting clinical trials.

^b^ PT involvement providing support as needed in collaboration with IEP team to maximize participation within classroom and access of school environment.

#### 3.1.1 Feeding

AS may present with hypotonia and failure to thrive (FTT) in infancy, but conservative treatment is usually sufficient. Sucking, chewing, and swallowing can be abnormal, including failure to breastfeed due to inadequate latching. Tongue protrusion and dyspraxia of swallowing and breathing during feeding contribute to FTT, and silent aspiration may occur. Early involvement of a feeding therapist is recommended. A feeding tube should be approached cautiously but considered when there is a history of aspiration pneumonia (Bindels‐de Heus et al., 2020; Glassman et al., 2017). Feeding tubes are required more commonly in individuals with a deletion. Choking remains a risk throughout life due to the combination of not chewing properly and stuffing the mouth.

#### 3.1.2 Growth

Height is usually normal (Bindels‐de Heus et al., 2020; Mertz et al., 2013; Tan et al., 2011). Disproportionately slow head growth may lead to microcephaly (≤−2 SDS) by age 2 years, particularly in those with deletion subtype. Absence of microcephaly is insufficient for rejection of a clinical suspicion of AS, especially in the non‐deletion subtypes (Williams et al., 2006).

#### 3.1.3 Hormones and puberty

The timing of puberty and menarche is normal. Regularity, duration, and severity of menses in females are typical (Kaskowitz et al., 2016). Hormonal alterations of puberty can affect epilepsy, anxiety, and behavior. Regulation of menses may be beneficial due to hygiene and impaired comprehension. Oral contraceptives and intramuscular progestogens are options, the latter with consideration of possible negative effect on bone health. Subdermal and intrauterine hormonal devices require anesthesia for initiation but are long‐acting, effective options. Discussing options with a gynecologist and/or endocrinologist is advised. Requests for permanent sterilization require formal consultation with experts in reproductive ethics as this is often not recommended (Albanese & Hopper, 2007; Kaskowitz et al., 2016). In adult males, public masturbation can be problematic and behavioral modifications are typically attempted initially (Smith, 2001). Use of medications such as selective serotonin reuptake inhibitors with decreased libido as a side effect may be helpful. Anti‐androgen therapy is not recommended. Fertility in individuals with AS is presumably normal. Their sociable disposition and cognitive impairment make them vulnerable to abuse (Smith, 2001). Regular Pap smear and gynecological exam under anesthesia is recommended per general guidelines, as individuals have a similar lifetime risk of uterine cancer to the general population.

#### 3.1.4 Immunization

Patients with AS have no evidence of immunodeficiency. Regular vaccination schedules are recommended, including vaccination for seasonal pathogens. COVID‐19 vaccination is recommended.

#### 3.1.5 Heat intolerance

Individuals with AS tend to overheat, presenting as flushing and diaphoresis. Predisposition to dehydration lowers seizure threshold. Breathable clothing is advised, including during sleep when overheating impacts sleep quality. Strategies to manage heat intolerance include limiting time outdoors when hot, encouraging intake of cold fluids, fans, water mist, cool rags, visors, sunglasses, and UV protective clothing.

#### 3.1.6 Toileting

In the literature, daytime urinary continence is reported in 35% but clinical experience suggests 75% (Radstaake et al., 2013). Regular toilet visits, diaper removal, positive reinforcement, and close monitoring contribute to success in continence (Radstaake et al., 2014). All toileting activities should be encouraged in the restroom including diaper changes. Strategies also include Applied Behavioral Analysis (ABA) to work on routine and reward‐based interventions (Radstaake et al., 2014; Warzak et al., 2016). Nighttime urinary continence is less common but facilitated by fluid restriction after dinner and toileting before bedtime. Please refer to Supplemental Figure I for guidance to an approach to toilet training (Figure S1).

#### 3.1.7 Dental

Brushing after teeth erupt is challenging. A double sided or electric toothbrush may be better tolerated. Mouthing, chewing, and gastroesophageal reflux disease (GERD) can cause erosion of the enamel. Drooling, causing continuous rinsing of the teeth, is a positive factor in dental hygiene. AS individuals may require sedation or anesthesia to conduct dental examinations and procedures, but this should not delay care (Khan et al., 2019).

#### 3.1.8 Anesthesia

Complications of anesthesia are rare, and typical management by the anesthesiologist is generally safe (Errando et al., 2007; Gardner et al., 2008; Kemper et al., 2010; Kim et al., 2010; Makris et al., 2018; Patil & Sindhakar, 2008; Rosado Fuentes et al., 2009; Warner et al., 2017; Witte et al., 2011). Specific intraoperative considerations associated with anesthesia include GABA receptor involvement in AS, since many intravenous and inhaled anesthetic agents modulate these receptors. Malignant bradyarrhythmias have been reported. Additional concerns include bradycardia due to increased vagal tone and delayed response to atropine (Gardner et al., 2008). A recent retrospective review suggests atypical responses to benzodiazepines, with one patient requiring flumazenil rescue; complications regarding airway management with one patient requiring videolaryngoscopic intubation; and a 2‐year‐old with intraoperative bronchospasm(Warner et al., 2017). Insufficient evidence exists to make specific anesthetic recommendations.

### 3.2 Epilepsy and movement disorders

Epilepsy occurs in up to 90% of individuals and is most common in those with a deletion (Bindels‐de Heus et al., 2020; Khan et al., 2019; Pelc et al., 2008; Williams, 2005; Williams et al., 2006). The mean age at onset of seizures was 1.7 years of age (range 3 months to 5 years) (Khan et al., 2019). Although at least 80–90% of children with deletions will develop epilepsy, the prevalence of seizures with mutations and UPD is up to 75%, and up to 50% for those with ICD (Thibert et al., 2009). Although the rate of seizures in the non‐deletion group is lower than the deletion group, 15% of seizures in the non‐deletion subtype had onset after age 5 years (equals 5% of all seizures, unpublished Rare Diseases Clinical Research Network).

For up to 1/3 of individuals, the first seizure will occur in the setting of a febrile illness (Thibert et al., 2013). Seizures tend to improve after puberty with a case series of 53 adults showing 65% resolution beginning at an average age of 16 years (Prasad et al., 2018). Seizure types at initial seizure presentation include myoclonic—25%, atonic—23%, generalized tonic‐clonic—21%, and atypical absences—12%. There are case reports of infantile spasms, but this seizure type is generally rare in AS. AS seizures are typically generalized, but up to 30% may also have focal seizures (Conant et al., 2009; Khan et al., 2019; Matsumoto et al., 1992; Pelc et al., 2008; Pollack et al., 2018; Prasad et al., 2018; Ranasinghe et al., 2015;9:^142^.; Sugimoto et al., 1992; Thibert et al., 2009, 2013).

Non‐convulsive status epilepticus (NCSE) is common, typically consisting of periods of decreased responsiveness or alertness, which may last hours to days, often with loss of developmental skills (Fujikawa et al., 2003; Ohtsuka et al., 2005; Yang et al., 2010). NCSE typically presents with few to no clinical seizures, so it may go unrecognized. While rates of NCSE have been reported in 50–90% of the population in older studies, a very recent study of 100 AS children followed over 7 years found that 19% had an episode of NCSE, while 5% experienced convulsive status epilepticus (Bindels‐de Heus et al., 2020). In a case series of 104 children in 2018, the rate was 20% (Worden et al., 2018). Rarely, NCSE will be accompanied by frequent myoclonic jerks, which is known as myoclonic status in non‐progressive encephalopathies (MSNPE)(Caraballo et al., 2007).

In addition to myoclonic seizures and MSNPE, individuals also have non‐epileptic myoclonus (NEM) most consistent with a movement disorder. Myoclonic seizures occur in younger age groups, whereas NEM occurs during adolescence and adulthood (Pollack et al., 2018). Both can be disabling, but myoclonic seizures have an EEG correlate and are generally controlled with medication as compared to NEM, which has no EEG correlate and can be disabling and refractory, though there is no alteration in consciousness (Casara et al., 1995; Pollack et al., 2018). Although evidence is lacking, experience suggests patients may benefit from medications effective in treating myoclonic seizures, such as levetiracetam, clobazam, and clonazepam, but the mainstay of therapy is to minimize triggers, such as poor sleep, GI dysfunction, and anxiety. A recent publication suggests a possible benefit of perampanel in the treatment of NEM (Kawano et al., 2020). In addition to NEM, AS movement disorders include tremor, ataxia, and possibly dystonia.

Evaluation for seizures includes an EEG, which is characterized by high amplitude slow (delta and theta) waves and a relative lack of normal background rhythms. High voltage (>300 microvolts) slowing with a “notched” delta (1–3 Hz) pattern is present in over 90% of individuals and provides a diagnostic clue (Korff et al., 2005; Laan et al., 1997; Sidorov et al., 2017; Thibert et al., 2013; Valente et al., 2003; Vendrame et al., 2012; Wang et al., 2005). Long‐term video EEG recording helps determine if a behavioral episode is a seizure or a non‐epileptic event. Blood chemistry and complete blood count testing exclude metabolic triggers and offer a baseline. Lumbar puncture and brain imaging are not indicated unless considering other etiologies.

**FIGURE 2:**
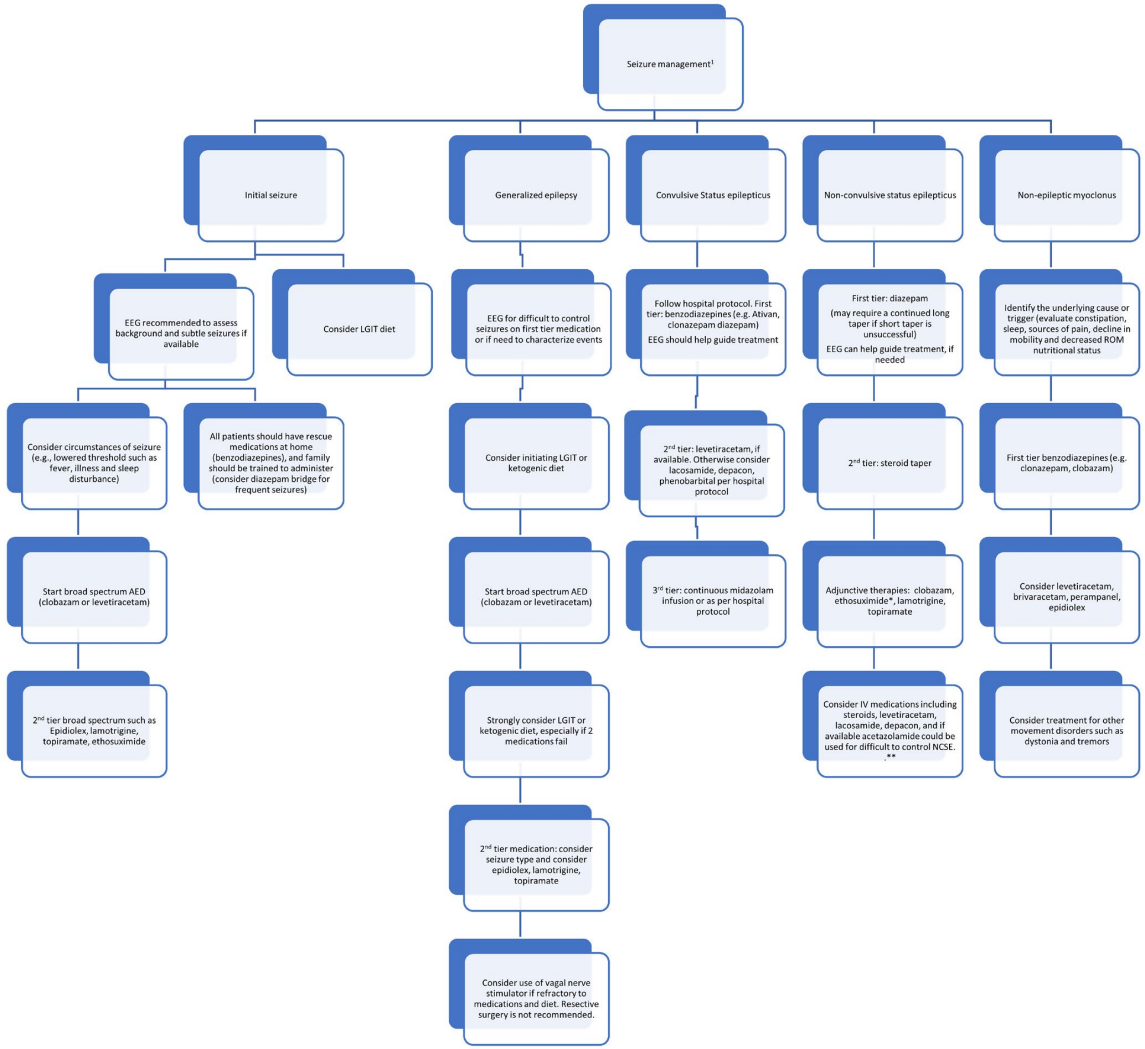
Management Algorithm for Seizures in Angelman Syndrome (AS). 1 Ask all patients about drug allergies. * Avoid phenytoin and phenobarbital for NCSE. All patients should have an emergency plan for management of seizures. Abbreviation ACD anticonvulsant drug; EEG electroencephalogram; LGIT low glycemic index therapy; NCSE nonconvulsive status epilepticus.

There are no comparative trials of the various anticonvulsant drugs (ACDs); thus, clinical practice is based on case series (Dan et al., 2007; Ostergaard & Balslev, 2001; Shaaya et al., 2016; Thibert et al., 2009). The ACDs likely to provide benefit with limited adverse effects include clobazam, levetiracetam, and clonazepam (Figure 2). The consensus recommendation is to treat with clobazam or levetiracetam as first line therapy and to consider dietary intervention, including a ketogenic diet (KD) (Evangeliou et al., 2010; Groesbeck et al., 2006) or low glycemic index therapy (LGIT)(Grocott et al., 2017; Shaaya et al., 2016; Thibert et al., 2012). KD is recommended for infants and children with feeding tubes, whereas for other children, LGIT is recommended as first‐line dietary therapy. If not completely effective, LGIT is converted to KD. Failure to control seizures with two medications warrants referral to an epileptologist. Since generalized seizures are prominent, broad spectrum ACDs are recommended. Phenobarbital, primidone, carbamazepine, phenytoin, and vigabatrin are contraindicated (Nolt et al., 2003). Parental reports of the effects of various forms of artisanal cannabidiol (CBD) for epilepsy are promising (unpublished); there is now an FDA‐approved CBD medication (Epidiolex). Although little evidence exists, this is a promising medication for seizures, and it may help NEM. The high rate of motor side effects caused by valproic acid indicates sparing use unless as a bridge medication or failure of other ACDs and diet (Shaaya et al., 2016). AS individuals should receive a prescription for a rescue medication such as rectal diazepam gel or intranasal midazolam for prolonged seizures. Interruption therapy for episodes of NCSE with diazepam divided 2–3 times daily with a taper over 5–7 days has benefit (Worden et al., 2018).

Seizure alarm devices may be considered but are not particularly helpful for subtle seizure types such as atonic and atypical absence seizures. Caregivers should be educated on seizure management, particularly on the recognition of NCSE. Some with AS are able to come off of ACDs eventually, but there are no clear guidelines. While ~65% of adults will become seizure‐free, many adolescents and adults will stay on ACDs, such as clobazam and clonazepam, to treat symptoms like sleep, anxiety, and NEM.

### 3.3 Gastrointestinal (Table S2)

#### 3.3.1 GERD/Vomiting

GERD is common and may persist or recur in adulthood (Bindels‐de Heus et al., 2020; Glassman et al., 2017). Signs include gagging, frequent swallowing, and pain or discomfort during and after feeding. In infants, therapy often starts with feeding in an upright position and keeping the child in this position for at least 30 mins post‐feeding. Adjustments in posture (e.g. side lying position), technique, and formula are helpful. Consideration should be given to thickening feeds and dietary modifications. Referral for intensive feeding therapy is recommended. Uncontrolled GERD may cause esophagitis and upper GI bleeding. In older individuals, vomiting can be a sign of gastroparesis. Back arching, poor sleep, gagging, regurgitation of food, and unexplained discomfort should prompt a trial of a proton pump inhibitor or H2 blocker. Prokinetic medication may treat refractory symptoms. Causes of vomiting include severe constipation, migraine variants, rumination, urinary tract infection, medication side effects, and anxiety. In some, vomiting becomes behavioral.

#### 3.3.2 Constipation

The prevalence of constipation is 80% and requires treatment (Bindels‐de Heus et al., 2020; Glassman et al., 2017). Constipation may contribute to increased seizures, sleep disturbance, and behavioral changes. The workup includes evaluation of fluid and fiber intake and adding dietary fiber when appropriate. Adding magnesium, probiotics, or medium‐chain triglyceride oil to the diet may help. Stool softeners are typically the next step after dietary changes, followed by stimulants. Refractory constipation may require intermittent or regular suppositories.

#### 3.3.3 Cyclic vomiting

Individuals may present with persistent vomiting episodes with serial vomiting and intervening symptom‐free periods (Glassman et al., 2017). This is distressing and often results in dehydration. The etiology may be a migraine variant, though anxiety and/or GERD may also play a role. In addition, allergies and/or acute illness causing congestion and post‐nasal drip may result in persistent emesis that requires antiemetics and/or fluid resuscitation. Treatment for chronic migraines, including amitriptyline, cyproheptadine, and topiramate, or treatments for anxiety and GERD as needed may be beneficial.

#### 3.3.4 Hyperphagia

Hyperphagia defined as inability to recognize satiety and related behaviors is recognized (Bindels‐de Heus et al., 2020; Carson et al., 2019; Fridman et al., 1998; Hanzlik et al., 2020; Kirkilionis et al., 1991). This may be characterized by an obsessive compulsive component and driven by times of anxiety, change in routine or transition in general. Those with uniparental disomy or ImpDs are at higher risk (Carson et al., 2019; Fridman et al., 1998). Management strategies include scheduled meal times with a visual schedule, the use of partitioned and smaller plates, and a diet with increased protein and decreased carbohydrates. Locking up food may be necessary. Food for rewards should be avoided. Prospective data from 100 parents of children with AS showed that 32% reported hyperphagia, varying from no intrinsic limit in eating to searching for food and eating nonfood items (Bindels‐de Heus et al., 2020). Up to 50% of adults have hyperphagia. Elevated body mass index (BMI) was associated with hyperphagia (Bindels‐de Heus et al., 2020).

#### 3.3.5 Food allergy and Eosinophilic Esophagitis

For children with difficult GI symptoms, food allergy testing should be considered. When allergy testing is negative, food sensitivities (e.g., dairy and gluten) may be present. An elimination diet, i.e., removing one food at a time for a short period (~2 weeks), may be helpful to identify the foods that worsen gas, bloating, and constipation. Some children with food allergies develop eosinophilic esophagitis (EoE), which typically requires an elimination diet and treatment with medications prescribed by gastroenterologists or allergists. In a case series from 2 clinics, 4% of 97 children with deletions or UPD had EoE, whereas none of the 23 children with mutations or ImpD had EoE (Glassman et al., 2017).

#### 3.3.6 Drooling

Drooling is caused by open‐mouth behavior, less frequent swallowing, or problems associated with the oral phase of swallowing. Unless there is accompanying aspiration, there is no indication for therapy, but social acceptance is a concern. Use of bibs and absorbent wristbands for mouth wiping are usually adequate. Stimulation of mouth closure and active swallowing can be done. Anticholinergic therapies such as glycopyrrolate or sublingual administration of atropine eyedrops (Norderyd et al., 2017) or parotid botulinum toxin injections may be employed, but the adverse effect of reducing saliva on dental health should considered (Gonzalez et al., 2017; Moller et al., 2015; Nordgarden et al., 2012; Pena et al., 2009; Reid et al., 2013). Side effects, like constipation, dry eyes, and thickened sputum, may prohibit these treatments.

#### 3.3.7 Dietary therapy

Research is necessary to further elucidate the optimal recommendations for dietary management. KD and LGIT are indicated for refractory epilepsy, but may also have other benefits (Evangeliou et al., 2010; Grocott et al., 2017; Groesbeck et al., 2006; Shaaya et al., 2016; Thibert et al., 2012). The classic KD is calculated in a ratio of grams (g) of fat to g of protein plus carbohydrate combined with 90% of calories from fat (Bergqvist, 2012; Evangeliou et al., 2010; Kossoff et al., 2002, 2018; Kwiterovich et al., 2003). A 3:1 or lower ratio can be used to increase protein or carbohydrate intake and is appropriate for diet initiation (Table S5)(Kossoff et al., 2018).

LGIT allows liberalization of carbohydrate intake to 40–60 g/day but regulates the type of carbohydrates, favoring those that produce small changes in blood glucose (glycemic index <50). The main side effects of KD/LGITs are gastrointestinal disturbances, hyperlipidemia (temporary), metabolic acidosis, and occasional renal calculi (Bergqvist, 2012; Kossoff et al., 2018). Children with treatment‐resistant epilepsy are at a high risk for poor bone health due to prolonged ACD exposure, direct and indirect effects of ACDs on calcium and vitamin D metabolism, and motor impairments that affect weight‐bearing. The combined effect of KD creating a high “acid load” via ketone bodies, alterations in vitamin D, and lowering of growth factors increases this risk (Bergqvist, 2012; Bergqvist et al., 2007, 2008). In the optimal clinical management recommendations of the KD Study Group, 48% of centers advocate screening with dual energy X‐ray absorptiometry (DEXA) scan in children to evaluate for osteopenia when on the KD for >2 years (Kossoff et al., 2018). Supplementation with vitamin D, calcium, and B vitamins should be provided at the recommended daily allowance (Vestergaard & Sayegh, 1966). A multivitamin with minerals is a universal recommendation (Makris et al., 2018). Levels of carnitine, selenium, magnesium, zinc, phosphorus, iron, and copper should be monitored along with beta‐hydroxybutyrate while on dietary therapy. Individuals with hyperphagia may benefit from more liberalized KDs such as Modified Atkins Diet or LGIT (Bindels‐de Heus et al., 2020).

### 3.4 Sleep

Sleep problems are present in up to 80% of individuals with AS (Williams, 2005), including problems with settling and insomnia, awakenings during the night, and early awakening. Besides a shorter sleep duration, sleep tends to be more fragmented (Bruni et al., 2004; Miano et al., 2004, 2005). Sleeping difficulties decrease with age, although many adolescents and adults continue to have disordered sleep and co‐sleep (Walz et al., 2005).

Poor sleep quality and diminished amounts of REM sleep negatively impact the regulation of behavior and worsen seizures (Dosier et al., 2017). Changes in behavior should prompt consideration of new onset sleep disorders. Epilepsy, ACDs, GERD, scoliosis, and constipation can further negatively impact sleep (Bindels‐de Heus et al., 2020). Individuals with AS should be screened for sleep problems and, if present, a detailed characterization of the sleep/wake schedule and routines should be investigated. Overnight polysomnography is indicated for individuals suspected of having sleep‐related breathing problems, nocturnal seizures, or unusual behavior during sleep. Given anxiety in unfamiliar environments, polysomnography may be challenging. A sleep diary and actigraphy (Braam et al., 2008; Iii, 2015) are non‐invasive means of characterizing sleep/wake patterns in the home environment. Video polysomnography may help to objectively observe sleep patterns.

For sleep disorders, treatment of contributing problems such as GERD, epilepsy, and behavioral issues including anxiety should be optimized. Specific sleep disorders such as obstructive sleep apnea (OSA) should be managed with the help of appropriate specialists. Sleep problems are initially treated with behavioral therapies and promoting sleep hygiene. Optimal sleep hygiene consists of a regular sleep‐wake rhythm, a bedtime routine, and a bedroom that is calm, cool, and dark (Hylkema & Vlaskamp, 2009). Individuals with AS benefit greatly from safety beds and we often recommend early referral for an enclosed bed in planning for transition from the crib. Behavioral therapies often consist of managing parent‐child interaction at night. Intensive coaching of parents can support behavioral interventions (Allen et al., 2013). Medicinal treatments targeting sleep difficulties are beneficial and often needed if behavioral therapies are ineffective. These include low‐dose melatonin (Braam et al., 2008; Egan et al., 2020; Galvan‐Manso et al., 2002; Paprocka et al., 2017; Takaesu et al., 2012; Zhdanova et al., 1999), alpha agonists (clonidine or guanfacine), benzodiazepines (clonazepam), gabapentin or pregabalin, antihistamines (promethazine or diphenhydramine), antidepressants (mirtazapine (Hanzlik et al., 2020) or trazodone), and in refractory cases antipsychotics (e.g. quetiapine (Wine et al., 2009), Figure 3).

**FIGURE 3:**
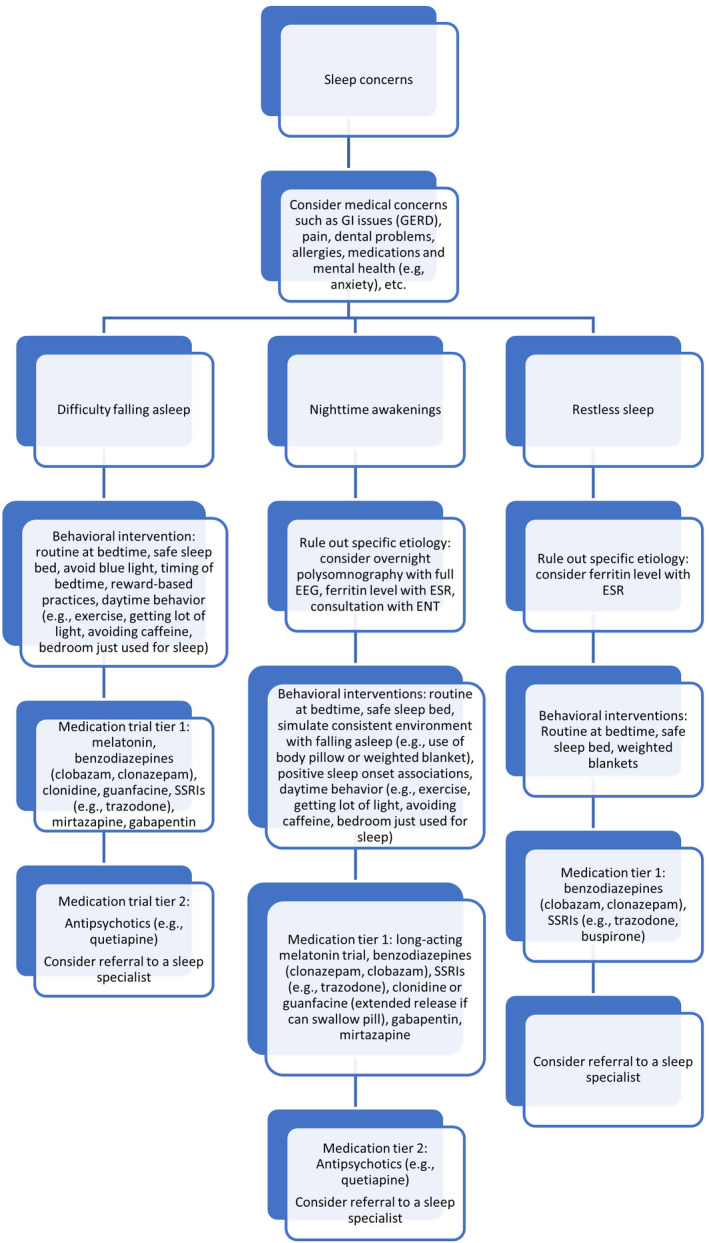
Management Algorithm for Sleep Disturbances in Angelman Syndrome (AS). Abbreviations: GI gastrointestinal; GERD gastroesophageal reflux disease, EEG electroencephalogram; ESR erythrocyte sedimentation rate; ENT otolaryngologist/ear, nose and throat specialist.

### 3.5 Cognitive and behavioral phenotypes

The neurodevelopmental profile of AS includes severe intellectual disability, global developmental delay, and lack of speech (Dagli et al., 2012). Studies have found significant delays across all domains, with cognitive skills trending below the 24–30 months developmental level, using the Bayley Scales of Infant Development (Gentile et al., 2010; Peters et al., 2004; Sahoo et al., 2007). Severity of cognitive delay correlates with molecular subtype (deletion is most severe), (Keute et al., 2020) but additional factors may contribute, such as underlying seizure control, selection of ACDs, access to habilitative therapies, and genetic background. Tables S2–S4 provide details of therapeutic interventions. At the time of delays, even prior to an official genetic diagnosis, referral to early interventional services for physical (PT), occupational (OT), and speech and language therapy (ST) are needed. Therapies should continue throughout life (Khan et al., 2019). Individuals with AS may benefit from alternative therapies in particular hydrotherapy especially due to their love of water. There is some evidence this improves behavior and social interaction in autism spectrum disorders (ASD) (Gueita‐Rodriguez et al., 2021; Mortimer et al., 2014). In addition it improves functional mobility of infant and toddlers (McManus & Kotelchuck, 2007), although the literature is varied (Getz et al., 2006). Hippotherapy benefits gross motor function and functional performance in individuals with neurodevelopmental disabilities (Park et al., 2014). It has been associated with improved body balance and posture and may have benefits for overall core strength (Matusiak‐Wieczorek et al., 2016). Little evidence‐base is available to suggest short periods of intensive physiotherapy; however, anecdotally programs such as Strengthening Program of Intensive Developmental Exercises and Activities for Reaching Maximal Potential or SPIDER therapy may be beneficial. More studies are required to recommend this to individuals with AS.

Frequent smiling and laughing resulting in an apparent happy disposition are hallmarks (Williams et al., 2006). Laughter may increase markedly with anxiety and some patients appear discomforted during pervasive bouts of laughter. Although rare, life‐threatening laughter is amenable to pharmacological treatment (Vanagt et al., 2005). Hyperactivity or hypermotoric behavior is consistently reported (Buntinx et al., 1995; Galvan‐Manso et al., 2002; Zori et al., 1992). Impulsivity is consistent with the level of intellectual disability (Barry et al., 2005). High rates of disruptive behaviors are reported (Arron et al., 2011; Larson et al., 2015; Sadhwani et al., 2019) during periods of excitement, while attempting to avoid nonpreferred tasks, or when seeking to maintain a caregiver's attention (Strachan et al., 2009). Such behaviors often appear to have communicative intent and may be triggered by internal states such as pain, fatigue, anxiety, or a preference for specific sensory input. A recent study using a modified anxiety questionnaire in 100 caregivers found high levels of distress with separation (Wheeler et al., 2019), which was lowest in individuals with a deletion subtype.

Individuals with AS have many features of ASDs such as repetitive behaviors and restricted/obsessive interests. This can include repetitive chewing/mouthing and stereotyped hand and body movements (Moss & Howlin, 2009). Sometimes, these movements are used to focus on a task or to reduce stimuli from the environment. Individuals with AS often respond to ABA therapy and referral is recommended for maladaptive behaviors and ASD features.

**FIGURE 4:**
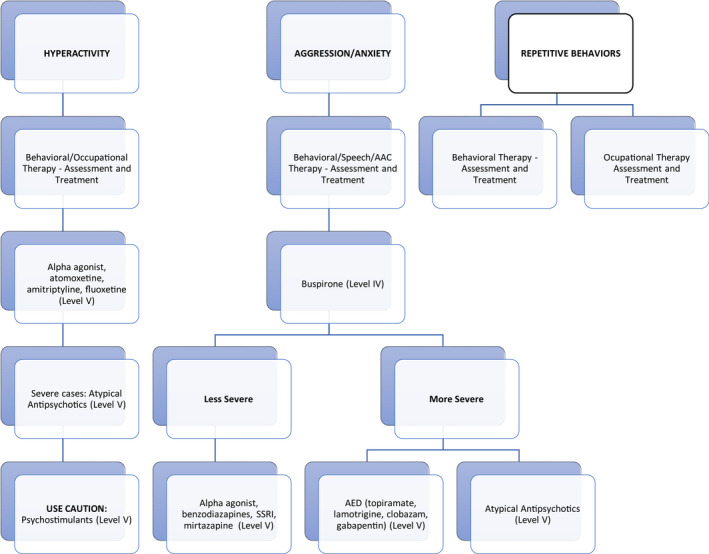
Management Algorithm for Behavior in Angelman Syndrome (AS). Abbreviation ACD anticonvulsant drug; AAC assistive and augmentative communication device; SSRI selective serotonin reuptake inhibitor.

New onset behavioral concerns or changes in sleep habits should prompt a thorough exploration for unrecognized medical illness/infection, constipation, dysmenorrhea, esophageal reflux, dental problems, scoliosis or post‐ictal confusion/sedation. Behavioral therapies, such as ABA, along with the use of an augmentative and assistive communication (AAC) device, should play an important role in the understanding and treatment of behavioral concerns, including reduction of problem behaviors and development of adaptive skills (Summers, 2012). Medication management is often needed (Figure 4).

Educators may have limited knowledge of AS. Developing an appropriate individualized education plan (IEP) capitalizing on the person's social skills while focusing on individualized 1:1 learning and instruction is critical to maximize educational potential as well as to improve communication and motor skills. Consistent with the Individuals with Disabilities Education Act, education should take place in the least restrictive environment. For some children with AS this might be in an integrated classroom alongside typically developing children, while for others it may be in a self‐contained classroom, or a combination of the two. Decisions about appropriate school/classroom placement should be arrived at through shared decision‐making between the school and family. Some individuals, particularly of the mosaic subtype achieve the ability to read and write to some degree (Hanzlik et al., 2020). The IEP should include intensive PT, OT, and ST (Table S2–S4). Parents, teachers, and caregivers should be trained and supported in the use of the AAC device with carryover from school to the home environment. If a child has behavioral challenges, as part of the IEP, families can request a functional behavioral assessment to assess for triggers and develop a management plan (Williams et al., 2006). A thorough AAC evaluation includes trials of at least 3 different AAC systems to effectively select the best device for the individual.

### 3.6 Ophthalmology (Table S2)

Evaluation by ophthalmology is recommended at diagnosis and annually (Dickinson et al., 1990). Strabismus is common (Khan et al., 2019; Michieletto et al., 2011; Williams, 2005). Conservative measures such as patching or corrective lenses are often difficult to implement because of an inability to cooperate with the treatment. Thirty percent of persons require strabismus surgery (Khan et al., 2019). In two small case series, persons with large angle exotropia showed excellent 6‐month post‐operative alignment after bilateral lateral rectus recessions (Mah et al., 2000; Ye et al., 2019).

Astigmatism is the most common refractive error. Almost all patients in an Italian study as well as 2 smaller series exhibited at least 1 diopter, with more than half showing potentially amblyogenic levels of astigmatism (>2 diopters) (Michieletto et al., 2011; Ye et al., 2019). While keratoconus has been described in adults, it is unclear whether this finding is associated with childhood astigmatism or behavioral eye rubbing (Larson et al., 2015). Further, in the Italian study, greater than 1 diopter of hyperopia or myopia was seen in 76% and 9% of the individuals, respectively (Michieletto et al., 2011). Thus, the majority of these children fit the general criteria for corrective lenses; however, tolerance of glasses is low, and rates of amblyopia have not been reported.

Assessment is often based on ophthalmic findings and visual behavior. In the literature, some individuals with AS have severe ophthalmic pathology such as optic and chorioretinal atrophy accounting for poor visual function (Rufa et al., 2003; Van Splunder et al., 2003). Clinically one of the first symptoms may be cortical visual impairment that improves with time (Micheletti et al., 2016). There is a wide spectrum of characteristics in cortical visual impairment that ranges from complete blindness to altered visual perception. Nystagmus has been reported in 11% of patients with cortical visual impairment (Huo et al., 1999). Consistent with this, 9% of individuals with AS exhibited nystagmus (Michieletto et al., 2011). Referral for OT/PT and vision/mobility services to maximize visual function is critical.

Oculocutaneous albinism due to a mutation in the *OCA2* gene located on the paternal copy of chromosome 15 should be considered in individuals with AS who have congenital nystagmus, iris hypopigmentation and translucency, reduced pigmentation of the retinal pigment epithelium, and foveal hypoplasia.. Identification of children with oculocutaneous albinism due to a deletion on chromosome 15 and a mutation on the other allele is important due to changes in management including regular use of sunscreen, management of low vision, regular ophthalmology follow up, and cancer screening.

### 3.7 Orthopedics

Lower bone density may occur in individuals with developmental delay compared to neurotypical individuals. Use of anti‐epileptic drugs and KD, immobility, decreased exposure to sunlight and late puberty are negative influencing factors (Srikanth et al., 2011). Low‐impact fractures may occur as a result. DEXA scan is recommended to assess bone health every 2 years depending on risk factors such as KD, non‐ambulatory individuals, ketogenic diet, history of >2 fractures without clear trauma, and age of onset of puberty. In females >65 years old screening should be implemented yearly. Vitamin D supplementation, stimulating daily physical activity (especially with vertical positioning), and 15–30 min of sunlight exposure/day are advised (Lin et al., 2015).

Hip dysplasia may occur due to external rotation of the legs along with decreased tone and delayed ambulation. Standard hip screening and radiographs in the frog leg position, particularly in non‐ambulatory children, should be performed, with orthopedic referral when indicated. Early PT is recommended to maintain or improve range of motion. The use of standers assist in increasing bone density.

At least 80% of children will achieve independent ambulation with an average age of onset of 3.7 years old for those with deletions (Williams et al., 2010). The gait pattern is associated with reduced step length versus cadence and continues throughout childhood despite adjustment for delayed ambulation onset (Grieco et al., 2018). Gait pattern and mobility change as individuals age; however, the commonly reported spasticity including dynamic contracture is not well characterized. Initial treatment should consist of PT and bracing to maintain range of motion and prevent static contractures. Gait analysis is recommended when considering orthopedic intervention, as many patients will develop maladaptive gait patterns which may worsen with inappropriate lengthening procedures (Larson et al., 2015). Larson reported that 10% of persons underwent tendon lengthening with varying results. Based on clinical observation, many patients develop limited ankle dorsiflexion with compensatory foot pronation and instability. This presentation can lead to a flexed knee gait pattern which is distinguished from a classic crouch gait in that passive ankle dorsiflexion is limited rather than excessive and accompanied by compensatory ankle pronation. In a sample of individuals, 62% presented with subluxed or pronated ankles (Williams et al., 2010; Zori et al., 1992), which can be treated with orthotics. If there is significant fixed angular deformity, corrective osteotomy remains controversial as recovery post‐procedure is often poor. Anecdotally, botulinum toxin injections may worsen gait and should be approached with caution due to the risk of exacerbating weakness and contributing to progression of a flexed knee gait pattern.

The incidence of scoliosis in children with AS is 10–30% (Smith, 2001; Zori et al., 1992) and in adults is 30–70% (Buntinx et al., 1995; Laan et al., 1996; Prasad et al., 2018). Most curves are thoracic, but up to 20% have increased lumbar lordosis associated with trunk weakness, increased anterior pelvic tilt, and crouch gait (Beckung et al., 2004). In adults, one study reported 21/22 patients had curves greater than 40 degrees (Guerrini et al., 2003). Standard screening with the forward bend test is appropriate for ambulatory patients, as well as orthopedic referral with radiographic monitoring. Curve assessment and treatment algorithms should follow those for typically developing children (Sewell et al., 2016). In non‐ambulatory patients, earlier use of thoracolumbosacral orthoses (TLSO) should be considered for promoting bimanual manipulation and social interaction. There are conflicting data regarding an association between ambulatory status and scoliosis progression, but there appears to be a female predominance (Laan et al., 1996; Larson et al., 2015; Smith, 2001). Continued monitoring of curve progression and effect on cardiopulmonary function and quality of life should guide intervention. There is only one study that has evaluated the benefit of surgical intervention for scoliosis (Sewell et al., 2016). While there was a small but significant absolute improvement in patient reported quality of life in the surgical group, there was a 60% complication rate. While this does not negate the indication for surgical treatment, frank pre‐operative risk‐benefit discussions should occur.

Orthopedic concerns as individuals with AS transition to adulthood include preserving range of motion and managing body weight. It is reported that over time hip and knee flexion contractures, scoliosis, and decreased stamina develop (Grieco et al., 2018). However, 64–75% of all adults with AS are able to walk independently. Plantar flexion contractures may also develop. By 13 years of age, up to 25% of AS patients will develop significant gait abnormalities which share features of crouch gait (Bindels‐de Heus et al., 2020). PT may be needed intermittently throughout life to address changes in function, gait, posture, range of motion, and strength (Table S3).

### 3.8 Clinical trials

Published clinical trials with negative results include: levodopa (Tan et al., 2018), minocycline (Grieco et al., 2014; Ruiz‐Antoran et al., 2018), and folic acid and betaine (Han et al., 2019; Keute et al., 2020; Peters et al., 2004, 2010) and betaine, metafolin, creatine, and vitamin B(12) (Bird et al., 2011). A phase 3 trial of gaboxadol (OV101) in children recently reported negative results (NCT04106557), despite promising phase 2 results in an adolescent and adult population (Bird et al., 2021). An exogenous ketone trial (Herber et al., 2020) recently showed safety and tolerability, improved stool consistency and a trend toward other benefits (Carson et al., 2021, NCT03644693). Phase I/II trials of antisense oligonucleotide therapies to activate the paternal copy of *UBE3A* are underway (NCT04428281, NCT04259281).

### 3.9 Adults with AS and transition of care

Adults have unique challenges: decline in mobility and a more sedentary lifestyle, NEM, GERD, constipation, anxiety, and behavioral concerns. Sleep, seizures, and hyperactivity may improve (Laan et al., 1996; Larson et al., 2015; Pelc et al., 2008). Transition to adulthood requires consideration of ongoing educational and therapeutic supports including access to behavioral therapy such as ABA and AAC device support, applying for disability, state‐specific waiver programs, and guardianship. An extensive IEP planning meeting should be done at 15–16 years old. There are several excellent resources such as https://www.gottransition.org/. Early planning includes use of community support resources (ARC and NORD) and patient support organizations (Foundation for Angelman Syndrome Therapeutics (FAST) and Angelman Syndrome Foundations (ASF)). Financial planning is critical and may include a special needs trust for the AS child/adult. Enrollment in vocational/recreational opportunities including adaptive sports or vocational training programs should be considered early. Utilization of transition clinics or the ASF clinical network for scheduled transition visits is advised. Preventive healthcare under anesthesia at generally recommended intervals is recommended.

### 3.10 Caregiver and family health

Caregivers are at high risk for experiencing negative consequences. Continued translationally oriented research is crucial to understand the specific needs of caregivers across the lifespan. Clinical specialists and providers should be aware that stressors are associated with caregiving, including negative impacts on social networks, family dynamics, and financial security, which lead to or exacerbate mental health and physical health challenges for caregivers. Providers can offer much needed relief such as respite care, which in turn can have long‐term positive impacts on the individual with AS and their support system as a whole (Adams et al., 2018; Bailey et al., 2009; Blucker et al., 2011; Buelow et al., 2006; Camfield et al., 2016; Didden et al., 2004; Falk et al., 2014; Miodrag & Peters, 2015; Murphy et al., 2007). The relationship of siblings is unique and requires family‐based approaches (Love et al., 2012). Regular individualized attention to siblings as an outlet to share concerns and challenges is important. Referral to support groups locally and nationally (FAST and ASF) with connection to other families is key to create support networks. Tools for siblings may include children's books and support groups.

## AUTHORS’ CONTRIBUTIONS

JD worked with AA to Chair the Steering Committee, develop the methods, invited the chairs of the committees, and provided oversight of the project. JD and her team completed the systematic review of the literature with input from 2 reviewers. JD compiled the final version of the manuscript, edited, and revised the manuscript. She was the chair of the Genetics Committee and completed meetings with the group. She attended group meetings of the other committees. MN drafted, edited, and revised the neurology section and served as a member of the Neurology Committee. JS served on the Behavioral Committee and edited the manuscript. LB edited and revised the manuscript. She provided mentorship for the Genetics Committee. KGCBB chaired the General Health Committee and revised and edited the manuscript. MJV was part of the General Health Committee and revised and edited the manuscript. MYDW edited and revised the manuscript and assisted with the Neurology and Sleep Committees. CN served on the Speech and Language Development Committee and revised and edited the accompanying section. MTHR chaired the Sleep Committee and revised and edited the manuscript. BMVIK served on the Physical Therapy Committee and edited and revised the accompanying section. SE chaired the Reproductive Health Committee and revised and edited the manuscript. MD served on the Reproductive Health Committee and revised and edited the manuscript. TK chaired the Toileting Committee. GDM and AK served on the Nutrition Committee and revised and edited the accompanying section. SN served on the Neurology Committee and revised and edited the manuscript. RT served on the Neurology Committee and revised and edited the manuscript. He met with JD and AA to review the final version. DG edited and revised the Sleep section of the manuscript. CK chaired the Behavioral Health Committee. He edited and revised the manuscript. KP and NS served on the Behavioral Health Committee and edited and revised the manuscript. AS Chaired the Development Committee and revised and edited the manuscript. HH edited and revised the manuscript. AW chaired the Caregiver Health Committee and revised and edited the manuscript. CW served on the Speech and Language Development Committee and revised and edited the accompanying section. MD chaired the Speech and Language Development Committee and revised and edited this section. AT chaired the Occupational Therapy Committee and revised and edited this section. EK and LD served on the Occupational Therapy Committee and revised and edited this section. KK chaired the Physical Therapy Committee and revised and edited the accompanying section. KA, CB, and NH served on the Physical Therapy Committee and revised and edited the accompanying section. JPG served on the Ophthalmology Committee and revised and edited the accompanying section. BLB chaired the Ophthalmology Committee and revised and edited this section. RCC served on the Orthopedics Committee and revised and edited this section. SH provided support to AA and revised and edited the manuscript. HGC chaired the Orthopedics Committee and revised and edited the accompanying section. KO served on the Caregiver Committee and revised and edited the accompanying section. ERJ served on the Steering Committee as a representative from the Angelman Syndrome Foundation and parent advocate and revised and edited the manuscript. AB served on the Steering Committee as a representative from the Foundation for Angelman Syndrome Therapeutics and parent advocate and revised and edited the manuscript. CAB served on the Genetic Committee and revised and edited the manuscript. CW served as a mentor to JD for the Genetics Committee and revised and edited the manuscript. AA was awarded funding through the Million Dollar Bike Ride. She served as part of the Neurology Committee.

## Supporting information

Fig S1

Table S1

Table S2

Table S3

Table S4

Table S5

## Data Availability

Data sharing not applicable – no new data generated.
