# Supplementary figures and images for: A multidisciplinary approach and consensus statement to establish standards of care for Angelman syndrome
Source: Mol Genet Genomic Med. 2022 Feb 11;10(3):e1843. doi: 10.1002/mgg3.1843 (PMC8922964; doi:10.1002/mgg3.1843)

## Slide 1
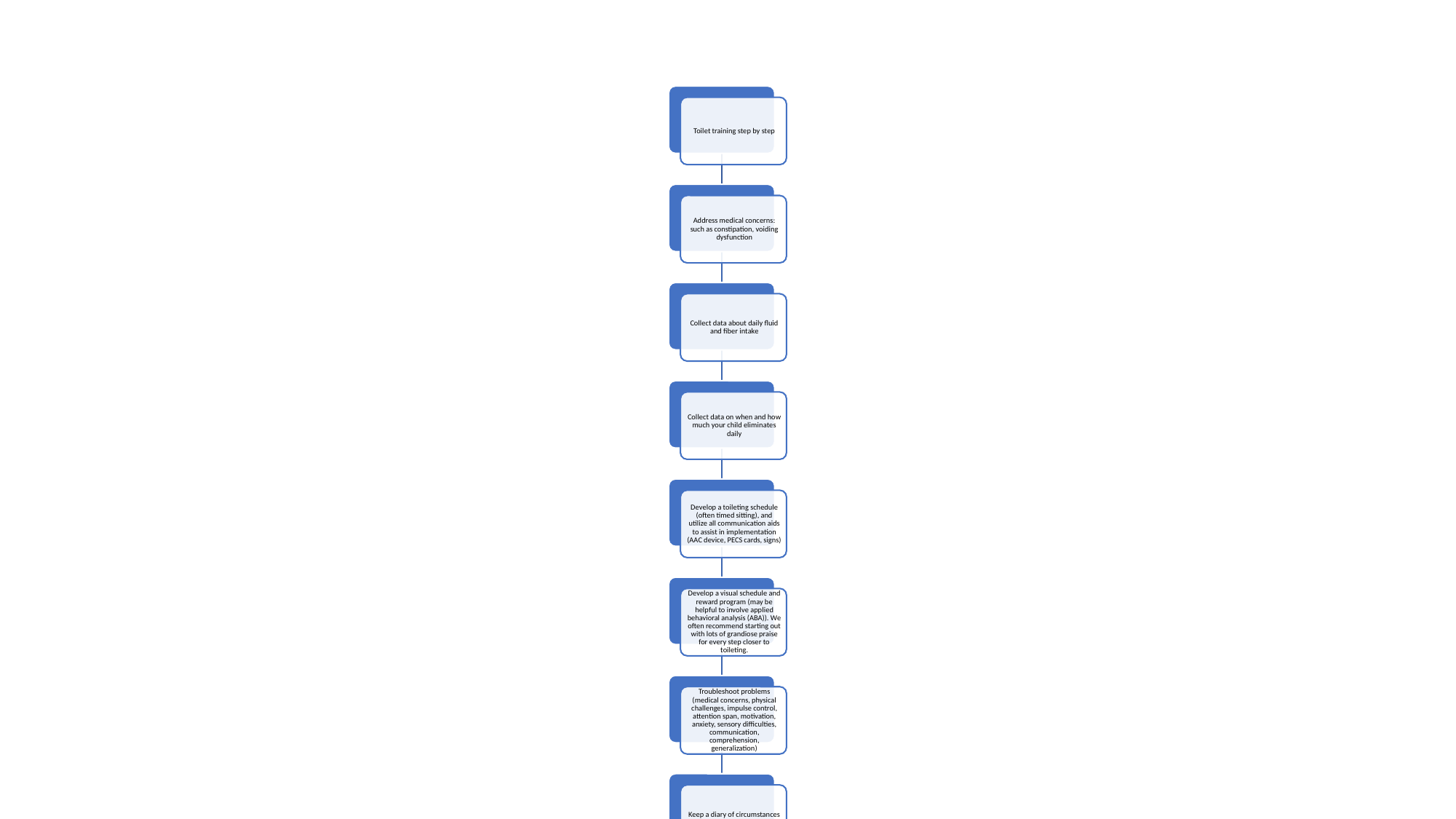

Supplement: Supplementary file 1 — Fig S1 [file MGG3-10-e1843-s006.pptx]
