# Supplementary material for: A multidisciplinary approach and consensus statement to establish standards of care for Angelman syndrome
Source: Mol Genet Genomic Med. 2022 Feb 11;10(3):e1843. doi: 10.1002/mgg3.1843 (PMC8922964; doi:10.1002/mgg3.1843)
Supplement: Supplementary file 2 — Table S1 [file MGG3-10-e1843-s003.docx]

Supplemental Table 1: Characteristic/treatment with recommendations.

| Characteristic | symptoms | Work- up | Management/treatment |
| --- | --- | --- | --- |
| Failure to thrive | Difficulty feeding may present in the newborn period and impact growth parameters | Feeding evaluation  Consideration of VFSS  Thyroid studies  Electrolytes  Complete Blood Count | Feeding therapy  Gastrostomy-tube is rarely needed in AS  Frequent follow up  Addition of high calorie supplements |
| Gastroesopha-geal reflux disease (GERD) | Frequent swallowing  Poor sleep  Arching  Pain/discomfort associated with feeding  Vomiting | pH probe  Endoscopy to rule out severe esophagitis/bleeding  Upper GI to rule out structural cause | Consider feeding in an upright position and keeping the child in the upright position for at least 30 minutes post-feeding  Trial of H2 blocker  Trial of PPI  Consider medication side effect for etiology of GERD |
| Vomiting | Intermittent or cyclic vomiting | Abdominal x-ray to screen for structural etiology  Urinalysis  Consider if anxiety-driven  Keep a diary of occurrences  Consider rumination  Consider migraines if cyclic | Treat underlying cause if identified  Consider empiric treatment if concern for anxiety, rumination or migraines  Consider blended diet |
| Constipation | Hard stools  Infrequent stools  Vomiting  Showing pain or discomfort  Loss of appetite  Worsening seizures  Poor sleep | Consider abdominal x-ray | Hospital admission if impacted for cleanout  Home cleanout: Miralax  64 grams in 16 ounces of water or electrolyte drink to start, 17 grams every 1-2 hours in 8 ounces of water or electrolyte drink  Avoid chronic constipation with natural supplements: magnesium, senna  Use macrogol upto 2 gram/kg once a day |
| Hyperphagia | Food seeking behavior, particularly high carbohydrate foods | Ferritin if consuming non-food products to rule out iron deficiency | Specialized diet: modified Atkins or LGIT  Routine mealtimes  Picture schedule  Trial of MCT (caution due to calories) |
| Drooling | Open mouth breathing, drooling |  | Sensory-based therapy  Therapy to improve muscle strength in facial muscle  Anticholinergic medication such a glycopyrrolate (monitor side effects such as constipation)  Parotid botox injection |
| Tremor | Often hand rhythmic movements | Consider MRI to rule out other known causes | Propranolol  L-dopa^9,11^ |
| Difficulty falling asleep |  |  | Bedtime routine  Avoid blue light close to bedtime  Avoid bedtimes too early in the evening  Trial of melatonin^12-15^  Clonidine |
| Nighttime awakening |  | Consider sleep study or EEG (could have worsening seizures) | Ensure conditions of falling asleep remain constant  Safe sleep bed  Trial of medications: trazodone, mirtazapine (can be used as needed), or quetiapine |
| Restless sleep | Consider sleep study | Check ferritin | Treat borderline or low ferritin with iron supplementation^16,17^ |
| Hyperactivity,impulsivity and inattention | Overly active and fidgety, unable to attend to a task, may manifest as aggression |  | ABA/behavioral therapy  Guanfacine or clonidine  Methylphenidate  Atomoxetine  Low dose neuroleptics (such as Risperdal) may help hyperactivity and impulsivity  Fluoxetine  Amitriptyline |
| Aggression | Includes self-injury  May be related to irritability or frustration with non-preferred demands  May be seen in periods of excitement  Attention-seeking | Consider medical causes: constipation, dysmenorrhea, GERD, dental concerns, scoliosis, seizures, worsening sleep  Consider anxiety (especially separation) | Behavioral consultation  Ensure options for communication (AAC)  ABA  Buspirone  Guanfacine, clonidine  Benzodiazepines  SSRI  Mirtazapine  Anti-epileptic medications (topiramate, lamotrigine, clobazam, gabapentin)  If severe, consider antipsychotic treatment (quetiapine preferred) |
| Repetitive behaviors | Repetitive chewing/mouthing  Stereotyped hand and body movements  Insistence on routine  Obsessional interests (water play) |  | Behavioral and occupational therapy  ABA |
| Strabismus | Exotropia (83%)  Esotropia (21%)  Vertical deviations (17%) | Referral to ophthalmology | Glasses and patching if significant refractive error^18-21^  Surgery for misalignment is greater than 10 diopters or poorly controlled^21^ |
| Refractive error | Astigmatism at least 1 diopter, with more than half showing potentially amblyogenic levels of astigmatism (>2 diopters).  Keratoconus |  | Glasses^19^ |
| Hypopigmentation | Affects iris and choroid  Not associated with foveal hypoplasia nor visual impairment |  | Possible role of OCA2 gene in 15q11.2q13 region, but hypopigmentation is not unique to deletion genotype^20,22-25^ |
| Visual function | Cortical visual impairment/decreased visual attention  Nystagmus (9%)  Rare optic atrophy or chorioretinal atrophy |  | Visual rehabilitation and therapies  Referral for occupational/physical therapy and vision/mobility services to maximize visual function^26^ |
| Developmental dysplasia of the hip |  | Standard hip screening by exam  Screening hip x-ray in the frog leg position, particularly in non-ambulatory children | Early intervention to improve ROM  Use of standers  Improve bimanual manipulation of objects and trunk muscle development if tolerated |
| Maladaptive gait pattern | Currently described as crouch  Subluxated pronated ankles | Formal gait analysis to avoid inappropriate lengthening procedures  Exam by experienced physician to identify decreased ROM versus spasticity | Physical therapy and bracing to maintain range of motion and prevent static contractures  Use caution with considering: botulinum toxin and tendon lengthening  Treat pronated ankles with night braces, shoe wedges of supramalleolar orthoses^27,28^ |
| Scoliosis | 10-30%^29,30^ and adults is 30-70%^31-33^  Most curves are thoracic but up to 20% have increased lumbar lordosis | Standard screening with the forward bend test | Curve assessment and treatment algorithms should follow typically developing children  In non-ambulatory patients, earlier use of thoracolumbosacral orthoses (TLSO) should be considered  Continued monitoring of curve progression and effect on cardiopulmonary function as well as quality of life should guide decision to intervene.  Surgical intervention (60% complication rate)^34,35^ |
| Osteopenia | rates near 16-20% | DEXA screening >3 fractures per year | Optimize vitamin D and calcium  Consider bisphosphonates in severe osteopenia (more than 2 fractures of long bones)  Maintain weight bearing^36^ |

References

1. Grocott OR, Herrington KS, Pfeifer HH, Thiele EA, Thibert RL. Low glycemic index treatment for seizure control in Angelman syndrome: A case series from the Center for Dietary Therapy of Epilepsy at the Massachusetts General Hospital. *Epilepsy Behav.* 2017;68:45-50.

2. Shaaya EA, Grocott OR, Laing O, Thibert RL. Seizure treatment in Angelman syndrome: A case series from the Angelman Syndrome Clinic at Massachusetts General Hospital. *Epilepsy Behav.* 2016;60:138-141.

3. Thibert RL, Pfeifer HH, Larson AM, et al. Low glycemic index treatment for seizures in Angelman syndrome. *Epilepsia.* 2012;53(9):1498-1502.

4. Ciarlone SL, Grieco JC, D'Agostino DP, Weeber EJ. Ketone ester supplementation attenuates seizure activity, and improves behavior and hippocampal synaptic plasticity in an Angelman syndrome mouse model. *Neurobiol Dis.* 2016;96:38-46.

5. Evangeliou A, Doulioglou V, Haidopoulou K, Aptouramani M, Spilioti M, Varlamis G. Ketogenic diet in a patient with Angelman syndrome. *Pediatr Int.* 2010;52(5):831-834.

6. Thibert RL, Conant KD, Braun EK, et al. Epilepsy in Angelman syndrome: a questionnaire-based assessment of the natural history and current treatment options. *Epilepsia.* 2009;50(11):2369-2376.

7. Worden L, Grocott O, Tourjee A, Chan F, Thibert R. Diazepam for outpatient treatment of nonconvulsive status epilepticus in pediatric patients with Angelman syndrome. *Epilepsy Behav.* 2018;82:74-80.

8. Goto M, Saito Y, Honda R, et al. Episodic tremors representing cortical myoclonus are characteristic in Angelman syndrome due to UBE3A mutations. *Brain Dev.* 2015;37(2):216-222.

9. Harbord M. Levodopa responsive Parkinsonism in adults with Angelman Syndrome. *J Clin Neurosci.* 2001;8(5):421-422.

10. Kawano O, Egawa K, Shiraishi H. Perampanel for nonepileptic myoclonus in Angelman syndrome. *Brain Dev.* 2020;42(5):389-392.

11. Tan WH, Bird LM, Sadhwani A, et al. A randomized controlled trial of levodopa in patients with Angelman syndrome. *Am J Med Genet A.* 2018;176(5):1099-1107.

12. Braam W, Didden R, Smits MG, Curfs LM. Melatonin for chronic insomnia in Angelman syndrome: a randomized placebo-controlled trial. *J Child Neurol.* 2008;23(6):649-654.

13. Braam W, Smits MG, Didden R, Korzilius H, Van Geijlswijk IM, Curfs LM. Exogenous melatonin for sleep problems in individuals with intellectual disability: a meta-analysis. *Dev Med Child Neurol.* 2009;51(5):340-349.

14. Takaesu Y, Komada Y, Inoue Y. Melatonin profile and its relation to circadian rhythm sleep disorders in Angelman syndrome patients. *Sleep Med.* 2012;13(9):1164-1170.

15. Zhdanova IV, Wurtman RJ, Wagstaff J. Effects of a low dose of melatonin on sleep in children with Angelman syndrome. *J Pediatr Endocrinol Metab.* 1999;12(1):57-67.

16. Herguner S, Kelesoglu FM, Tanidir C, Copur M. Ferritin and iron levels in children with autistic disorder. *Eur J Pediatr.* 2012;171(1):143-146.

17. Ryan CS, Edlund W, Mandrekar J, Wong-Kisiel LC, Gavrilova RH, Kotagal S. Iron Deficiency and Its Role in Sleep Disruption in Patients With Angelman Syndrome. *J Child Neurol.* 2020:883073820941755.

18. Dagli AI, Mueller J, Williams CA. Angelman Syndrome. In: Adam MP, Ardinger HH, Pagon RA, et al., eds. *GeneReviews((R)).* Seattle (WA)1993.

19. Mah ML, Wallace DK, Powell CM. Ophthalmic manifestations of Angelman syndrome. *J AAPOS.* 2000;4(4):248-249.

20. Michieletto P, Bonanni P, Pensiero S. Ophthalmic findings in Angelman syndrome. *J AAPOS.* 2011;15(2):158-161.

21. Ye H, Lan X, Liu Q, et al. Ocular findings and strabismus surgery outcomes in Chinese children with Angelman syndrome: Three case reports. *Medicine (Baltimore).* 2019;98(51):e18077.

22. Dickinson AJ, Fielder AR, Young ID, Duckett DP. Ocular findings in Angelman's (happy puppet) syndrome. *Ophthalmic Paediatr Genet.* 1990;11(1):1-6.

23. Fridman C, Hosomi N, Varela MC, Souza AH, Fukai K, Koiffmann CP. Angelman syndrome associated with oculocutaneous albinism due to an intragenic deletion of the P gene. *Am J Med Genet A.* 2003;119A(2):180-183.

24. Fukiyama Y, Tonari M, Matsuo J, et al. A Case of Fundus Oculi Albinoticus Diagnosed as Angelman Syndrome by Genetic Testing. *Case Rep Ophthalmol.* 2018;9(1):102-107.

25. King RA, Wiesner GL, Townsend D, White JG. Hypopigmentation in Angelman syndrome. *Am J Med Genet.* 1993;46(1):40-44.

26. Van Splunder J, Stilma JS, Evenhuis HM. Visual performance in specific syndromes associated with intellectual disability. *Eur J Ophthalmol.* 2003;13(6):566-574.

27. Bindels-de Heus K, Mous SE, Ten Hooven-Radstaake M, et al. An overview of health issues and development in a large clinical cohort of children with Angelman syndrome. *Am J Med Genet A.* 2020;182(1):53-63.

28. Bonanni P, Gobbo A, Nappi S, et al. Functioning and disability in patients with Angelman syndrome: utility of the International Classification of functioning disability and health, children and youth adaptation framework. *Disabil Rehabil.* 2009;31 Suppl 1:S121-127.

29. Zori RT, Hendrickson J, Woolven S, Whidden EM, Gray B, Williams CA. Angelman syndrome: clinical profile. *J Child Neurol.* 1992;7(3):270-280.

30. Smith JC. Angelman syndrome: evolution of the phenotype in adolescents and adults. *Dev Med Child Neurol.* 2001;43(7):476-480.

31. Buntinx IM, Hennekam RC, Brouwer OF, et al. Clinical profile of Angelman syndrome at different ages. *Am J Med Genet.* 1995;56(2):176-183.

32. Laan LA, den Boer AT, Hennekam RC, Renier WO, Brouwer OF. Angelman syndrome in adulthood. *Am J Med Genet.* 1996;66(3):356-360.

33. Prasad A, Grocott O, Parkin K, Larson A, Thibert RL. Angelman syndrome in adolescence and adulthood: A retrospective chart review of 53 cases. *Am J Med Genet A.* 2018;176(6):1327-1334.

34. Larson AM, Shinnick JE, Shaaya EA, Thiele EA, Thibert RL. Angelman syndrome in adulthood. *Am J Med Genet A.* 2015;167A(2):331-344.

35. Sewell MD, Wallace C, Gibson A, et al. A retrospective review to assess whether spinal fusion and scoliosis correction improved activity and participation for children with Angelman syndrome. *Dev Neurorehabil.* 2016;19(5):315-320.

36. Coppola G, Verrotti A, Mainolfi C, et al. Bone mineral density in angelman syndrome. *Pediatr Neurol.* 2007;37(6):411-416.
