# Supplementary material for: A multidisciplinary approach and consensus statement to establish standards of care for Angelman syndrome
Source: Mol Genet Genomic Med. 2022 Feb 11;10(3):e1843. doi: 10.1002/mgg3.1843 (PMC8922964; doi:10.1002/mgg3.1843)
Supplement: Supplementary file 3 — Table S2 [file MGG3-10-e1843-s002.docx]

**Supplemental Table 2: Speech and Language Standards of Care for Individuals with AS**

| **Speech Sound and Language Development**  Currently, few individuals with AS use spoken language as their primary means of expressive communication, as they typically present with a severe speech impairment with minimal or no use of words.^1-3^   - Vocal development is limited; less babble and other vocalizations^4^ - Limited repertoire of sounds; primarily open vowel sounds and sounds of pleasure/displeasure^5^ - Some differences in speech acquisition amongst various genetic mechanisms of AS:   - Individuals with a deletion of the UBE3A gene on chromosome 15 generally demonstrate the least vocal development, with more than 70% having no functional verbal speech ^6^   - Those with AS due to uniparental disomy (UPD) have been noted to produce up to 20 spoken words and word approximations^7,8^   - Individuals with AS due to a mosaic imprinting center defect (ICD) tend to use more words than other phenotypes, with some speaking in sentences ^7,8^ - Due to significant challenges with motor planning and coordination of motor skills, the limited production of sounds and spoken words is likely due to apraxia of speech^4^ - Receptive language skills are better developed than expressive language abilities^1,9-15^   **Communication Skills**  Individuals with AS communicate for variety of pragmatic purposes^15,16^ using a range of means, including body movements, gestures (e.g., reaching, pointing, clapping, waving), eye gaze, facial expressions, sign language, and a range of AAC systems (e.g., object symbols, photographs, picture symbols, picture communication boards and books, and speech generating communication devices)   - Most often communicate to refuse, request, and interact socially; less likely to communicate to exchange information (i.e., ask/answer questions, label, comment)^17,18^ - Due to motor challenges, use of sign language as primary mean of communication is unfeasible   Functional communication is critical; if an individual’s communication system is not effective or efficient, it is probable, if not addressed, behavior problems (e.g., hitting, grabbing, biting, pulling hair) may occur   - Providing individuals with conventional and symbolic means of communicating functionally and effectively is vital - Outlined as a basic human right in the Communication Bill of Rights by the National Joint Committee for the Communication Needs of Persons with Severe Disabilities (NJC)^19^, it is essential to appropriately assess an individual’s skills and provide meaningful intervention for the individual as well as their caregivers - Individuals with Angelman syndrome (AS) should be referred to as having “complex communication needs” (CCN) rather than using the term “nonverbal” |
| --- |
| **Communication Assessment**  Standardized measures do not accurately describe the communication skills of an individual with AS; rather, inventory an individual’s expressive communication functions and means using useful tools for planning goals and strategies for intervention, which can also be used to track progress over time, such as:   - *Communication Matrix^20^* - *Pragmatics Profile of Everyday Communication Skills in Children*   **Assessment for AAC**  A comprehensive evaluation for an augmentative and alternative communication (AAC) system is an ongoing, dynamic process, which may occur over weeks or even months.   - It is important to work with a professional or team of professionals who specialize in AAC to ensure an individual trials and obtains an appropriate AAC system, based on individualized feature matching, as there is no “one-size-fits all” device, and no one system is appropriate for every individual with AS. |
| **Intervention and Treatment Strategies**  In general, a multi-modal, or total, communication approach is recommended with individuals with AS, as they may use a variety of communication modes (vocalizations, signs, gestures, pictures, spoken words, AAC) to support functional communication   - Spontaneous communication is the aim of intervention, ultimately.   Speech Sound and Spoken Language Treatment  Although no research has demonstrated specific interventions targeting speech production to work for individuals with AS, several therapeutic approaches to promote speech sound development and spoken language have been shown to be effective for individuals with apraxia of speech^20^   - Integral Stimulation/Dynamic Temporal and Tactile Cueing (DTTC)^21^ - PROMPTs for Restructuring Oral Muscular Phonetic Targets (PROMPT)^22^   Augmentative and Alternative Communication (AAC)  AAC has been suggested as a beneficial therapeutic approach for individuals with AS therefore, most interventions concentrate on implementing AAC with individuals with AS.  Modeling AAC (Aided Language Stimulation/Input) and Communication Partner Training   - Modeling AAC, or using aided language stimulation/input, promotes AAC use in children with CCNs^23^ and should be considered evidenced-based practice^24^ - Aided language stimulation, or input, is a strategy where the communication partner pairs their spoken communication with modeling AAC during every day communicative contexts and interactions^25-31^; the receptive language input the AAC learner receives is not only spoken but simultaneously modeled in the AAC language system the individual is being taught to use expressively - While the communication partner models, no demands are put on the AAC learner/user - Supporting and training communication partners within those AAC trajectories is essential^32^   Promoting Literacy  It is imperative for intervention to focus on developing literacy skills using comprehensive emergent literacy instruction (i.e., modeling AAC, shared reading, independent reading, shared writing, independent writing, alphabet and phonological awareness)^33^   - Alphabet knowledge and phonological awareness are developed through both shared and independent reading and writing opportunities; examples include learning to recognize the first letter of one’s name, reading and writing alphabet books, rhyming, and both blending and segmenting words - Shared reading promotes interactive opportunities to make connections between text and real-life experiences, which builds background and world knowledge; examples of shared reading strategies include dialogic reading, thinking and commenting aloud, and print referencing^33-35^ - Using comprehensive literacy intervention strategies with individuals with AS provides an opportunity to learn multiple modalities of language, combining listening, expressing, reading, and writing, and a deeper development and understanding of language - The ability to read and write allows a person to learn and express infinite ideas - Multiple resources and programs are available to support the development of literacy skills for individuals with CCN |
| **Barriers**  Individuals with AS experience a variety of barriers which impede their access to communicating personal, medical, and educational needs   - Often these barriers are a result of the attitudes of professionals, parents, as well as the community^36^ - For an individual with AS, they extend to inclusion in general education, workplace and community settings, access to the same life experiences as peers (including physical, linguistic, vision, hearing, and other sensory deficits), access to repeated exposures (including repeated storytelling and educational instruction), as well as access and opportunity to use a robust vocabulary system, which includes access to a keyboard^26^ - Additional external barriers include limited access to knowledgeable providers (including physicians, specialists, educators, and therapists) due to geography, financial limitations   Barriers Specific to AAC   - Aided AAC system abandonment is common within two weeks to two years of receipt^27^; this can be attributed to limited or inconsistent acceptance by caregivers and providers, who may limit access to AAC systems - Within the field of AAC, research has shown: “(1) parents were influenced by the attitudes and experience of professionals; (2) parents did not feel supported by SLPs; (3) communication between stakeholders was not effective; and (4) parents had difficulties using AAC without a supportive community”^27^ |
| **Summary**  Individuals with AS use a wide variety of communication tools to communicate for a variety of purposes. As communication is a basic human right^28^, the need for individuals with AS to access communication through any and all modalities is paramount to their quality of life with not only their immediate family but their extended community as well. Therefore, collaboration amongst the individual’s social circle of communication partners, including family, peers, therapists, teachers, support staff, and employers to support acceptance and access to AAC is required to promote consistency across settings. |
| **Resources for Families, Caregivers, Teachers, Interventionists, and Support Staff**   - The Angelman Syndrome Foundation (ASF) Communication Training Series houses a wealth of free webinars by experts in the field AAC and literacy development: <https://www.angelman.org/resources-education/communication-training-series/> - The Angelman Academy is a collection of for-purchase resources and courses related to AAC and education for individuals with AS: <https://angelmanacademy.org> - The Center for Literacy and Disabilities Studies at UNC-Chapel Hill has created many resources for promoting AAC and literacy instruction: <https://www.med.unc.edu/ahs/clds/>   - Project Core offers free core language board downloads, free professional developmental modules about modeling core language and promoting literacy throughout the day, and many other useful resources. <http://www.project-core.com/>   - The Dynamic Learning Maps Professional Development Site has many resources and supports <http://dlmpd.com>   - Creating books about an individual (including family, interests, activities) encourages participation in shared reading activities; Tar Heel Reader is a website where one can upload pictures and write books pertaining to the pictures: <http://tarheelreader.org> - The Literacy Instruction for Students with Significant Disabilities website offers information and evidence-based strategies for promoting literacy skills for individuals with CCNs: <http://literacyforallinstruction.ca/> - Accessible Literacy Learning (ALL) Reading Program is a curriculum for teaching literacy skills to individuals with CCNs: <https://aacliteracy.psu.edu/> |

1. Clayton-Smith J. Clinical research on Angelman syndrome in the United Kingdom: observations on 82 affected individuals. *Am J Med Genet.* 1993;46(1):12-15.

2. Dan B. The paediatrician's role in support groups for rare diseases. *Acta Paediatr.* 2008;97(11):1510-1511.

3. Williams CA, Driscoll DJ, Dagli AI. Clinical and genetic aspects of Angelman syndrome. *Genet Med.* 2010;12(7):385-395.

4. Guerrini R, Carrozzo R, Rinaldi R, Bonanni P. Angelman syndrome: etiology, clinical features, diagnosis, and management of symptoms. *Paediatr Drugs.* 2003;5(10):647-661.

5. Grieco JC, Gouelle A, Weeber EJ. Identification of spatiotemporal gait parameters and pressure-related characteristics in children with Angelman syndrome: A pilot study. *J Appl Res Intellect Disabil.* 2018;31(6):1219-1224.

6. Lossie AC, Whitney MM, Amidon D, et al. Distinct phenotypes distinguish the molecular classes of Angelman syndrome. *J Med Genet.* 2001;38(12):834-845.

7. Nazlican H, Zeschnigk M, Claussen U, et al. Somatic mosaicism in patients with Angelman syndrome and an imprinting defect. *Hum Mol Genet.* 2004;13(21):2547-2555.

8. Carson RP, Bird L, Childers AK, Wheeler F, Duis J. Preserved expressive language as a phenotypic determinant of Mosaic Angelman Syndrome. *Mol Genet Genomic Med.* 2019;7(9):e837.

9. Jolleff N, Ryan MM. Communication development in Angelman's syndrome. *Arch Dis Child.* 1993;69(1):148-150.

10. Anderson RT. Learning an invented inflectional morpheme in Spanish by children with typical language skills and with specific language impairment (SLI). *Int J Lang Commun Disord.* 2001;36(1):1-19.

11. Duker PC, van Driel S, van de Bercken J. Communication profiles of individuals with Down's syndrome, Angelman syndrome and pervasive developmental disorder. *J Intellect Disabil Res.* 2002;46(Pt 1):35-40.

12. Trillingsgaard A, JR OS. Autism in Angelman syndrome: an exploration of comorbidity. *Autism.* 2004;8(2):163-174.

13. Gentile JK, Tan WH, Horowitz LT, et al. A neurodevelopmental survey of Angelman syndrome with genotype-phenotype correlations. *J Dev Behav Pediatr.* 2010;31(7):592-601.

14. Granild Bie Mertz L, Christensen R, Vogel I, Hertz JM, Ostergaard JR. Epilepsy and cataplexy in Angelman syndrome. Genotype-phenotype correlations. *Res Dev Disabil.* 2016;56:177-182.

15. Pearson E, Wilde L, Heald M, Royston R, Oliver C. Communication in Angelman syndrome: a scoping review. *Dev Med Child Neurol.* 2019;61(11):1266-1274.

16. Calculator SN. Parents' reports of patterns of use and exposure to practices associated with AAC acceptance by individuals with Angelman syndrome. *Augment Altern Commun.* 2013;29(2):146-158.

17. Wheeler AC, Sacco P, Cabo R. Unmet clinical needs and burden in Angelman syndrome: a review of the literature. *Orphanet J Rare Dis.* 2017;12(1):164.

18. Didden R, Korzilius H, Duker P, Curfs L. Communicative functioning in individuals with Angelman syndrome: a comparative study. *Disabil Rehabil.* 2004;26(21-22):1263-1267.

19. Brady NC, Bruce S, Goldman A, et al. Communication Services and Supports for Individuals With Severe Disabilities: Guidance for Assessment and Intervention. *Am J Intellect Dev Disabil.* 2016;121(2):121-138.

20. Rowland C, Fried-Oken M. Communication Matrix: A clinical and research assessment tool targeting children with severe communication disorders. *J Pediatr Rehabil Med.* 2010;3(4):319-329.

21. Strand EA. Dynamic Temporal and Tactile Cueing: A Treatment Strategy for Childhood Apraxia of Speech. *Am J Speech Lang Pathol.* 2020;29(1):30-48.

22. Dale PS, Hayden DA. Treating speech subsystems in childhood apraxia of speech with tactual input: the PROMPT approach. *Am J Speech Lang Pathol.* 2013;22(4):644-661.

23. Murray E, McCabe P, Ballard KJ. A systematic review of treatment outcomes for children with childhood apraxia of speech. *Am J Speech Lang Pathol.* 2014;23(3):486-504.

24. Lynch TJ, Erickson BJ, Miller DR, Finkelstein RR. ABI5-binding proteins (AFPs) alter transcription of ABA-induced genes via a variety of interactions with chromatin modifiers. *Plant Mol Biol.* 2017;93(4-5):403-418.

25. Sennott C, Reniers G, Gomez-Olive FX, Menken J. Premarital Births and Union Formation in Rural South Africa. *Int Perspect Sex Reprod Health.* 2016;42(4):187-196.

26. Beck AR, Stoner JB, Dennis ML. An investigation of aided language stimulation: does it increase AAC use with adults with developmental disabilities and complex communication needs? *Augment Altern Commun.* 2009;25(1):42-54.

27. Biggs EE, Carter EW, Gilson CB. Systematic Review of Interventions Involving Aided AAC Modeling for Children With Complex Communication Needs. *Am J Intellect Dev Disabil.* 2018;123(5):443-473.

28. Binger C, Maguire-Marshall M, Kent-Walsh J. Using aided AAC models, recasts, and contrastive targets to teach grammatical morphemes to children who use AAC. *J Speech Lang Hear Res.* 2011;54(1):160-176.

29. Gevarter C, Zamora C. Naturalistic Speech-Generating Device Interventions for Children With Complex Communication Needs: A Systematic Review of Single-Subject Studies. *Am J Speech Lang Pathol.* 2018;27(3):1073-1090.

30. Dada S, Alant E. The effect of aided language stimulation on vocabulary acquisition in children with little or no functional speech. *Am J Speech Lang Pathol.* 2009;18(1):50-64.

31. Harris MD, Reichle J. The impact of aided language stimulation on symbol comprehension and production in children with moderate cognitive disabilities. *Am J Speech Lang Pathol.* 2004;13(2):155-167.

32. Light J, McNaughton D. Designing AAC Research and Intervention to Improve Outcomes for Individuals with Complex Communication Needs. *Augment Altern Commun.* 2015;31(2):85-96.

33. Erickson A, Waldhaus K, David T, et al. Plasma treated with amotosalen and ultraviolet A light retains activity for hemostasis after 5 days post-thaw storage at 1 to 6(o) C. *Transfusion.* 2017;57(4):997-1006.

34. Justice LM, Ezell HK. Print referencing: an emergent literacy enhancement strategy and its clinical applications. *Lang Speech Hear Serv Sch.* 2004;35(2):185-193.

35. Zucker TA, Justice LM, Piasta SB. Prekindergarten teachers' verbal references to print during classroom-based, large-group shared reading. *Lang Speech Hear Serv Sch.* 2009;40(4):376-392.

36. Moorcroft A, Scarinci N, Meyer C. Speech pathologist perspectives on the acceptance versus rejection or abandonment of AAC systems for children with complex communication needs. *Augment Altern Commun.* 2019;35(3):193-204.
