# Supplementary material for: A multidisciplinary approach and consensus statement to establish standards of care for Angelman syndrome
Source: Mol Genet Genomic Med. 2022 Feb 11;10(3):e1843. doi: 10.1002/mgg3.1843 (PMC8922964; doi:10.1002/mgg3.1843)
Supplement: Supplementary file 4 — Table S3 [file MGG3-10-e1843-s004.docx]

Supplemental Table 3: Physical Therapy:

| **Age** | **Notable Characteristics** | **Intervention** |
| --- | --- | --- |
| Age of diagnosis-2 | -Delayed motor milestones  -Hypotonia (generalized)  -Hyperexcitable | -Regular intervention recommended depending on goals and priority of child and family.  -Encourage transitions in a natural developmental progression  -Strengthening exercises with focus on head control, trunk, and extremities  -Trunk support for sitting or standing activities may be needed to reduce risk of scoliosis  -Orthotics (AFOs, SMOs, UCBs, FOs) for standing and walking  - Consider equipment such as stander, gait-trainer, reverse walker  -Referral as needed to orthopedic provider for spine, hip, and foot/ankle management |
| Toddler (2-4) | -Delayed motor milestones  -Emerging ambulation  -Ataxia | -Regular intervention recommended depending on goals and priority of child and family  -Continue transitions in a natural developmental progression  -Strengthening exercises with focus on trunk and extremities  -Orthotics (AFOs, SMOs, UCBs, FOs)  -Consider equipment such as stander, gait-trainer, reverse walker  -Consider aquatherapy and hippotherapy  -Body suit/compression garments to help with posture and alignment  -Referral as needed to orthopedic provider for spine, hip, and foot/ankle management |
| Childhood (4-8) | -Delayed motor milestones  -Hypotonia  -Ataxia  -Most individuals are walking, and may need supports due to hypotonia  -Increasing need for foot/ankle support  - Emerging risk of lower extremity contracture | -Variable frequency of PT depending on goals and priorities of family (Note: maintaining regular physical activity helps with continued strengthening and joint ROM)  -Maintenance of ROM, specifically ankle dorsiflexion (with neutral midfoot), knee extension, and hip extension  -Strengthening program for proximal and distal muscle groups  -Orthotics (AFOs, SMOs, UCBs, FOs)  -Initiate regular exercise through community with adaptive sports and exercise classes  -Referral as needed to orthopedic provider for spine, hip, and foot/ankle management  -Consider aquatherapy, hippotherapy, and SPIDER therapy  -Avoid use of botulinum toxin (“botox”) |
| Pre-pubertal (8-12 years old) | -Monitoring for changes in gait and endurance  -During this time it is possible we may see flexed knee gait emerge, characterized by increased anterior pelvic tilt, knee flexion, and ankle pronation  -Contracture development may be confused with increased muscle tone and should be carefully assessed | -Consider gait study  -Intermittent PT to address changes in function, gait, ROM, and/or strength, and to promote healthy, active lifestyle  -Trunk strengthening/stability  -Orthotics (AFOs, SMOs, UCBs, FOs)  -Regular strength training  -Continue regular exercise through community with adaptive sports and exercise classes  -Focus on ROM, specifically ankle dorsiflexion (with neutral midfoot), knee extension and hip extension  -Balance activities  -Referral as needed to orthopedic provider for spine, hip, and foot/ankle management  -Consider aquatherapy, hippotherapy, and other strengthening activities.  -Avoid use of botulinum toxin (“botox”) |
| Pubertal (12-18 years old) | -Frequent decline in gait and endurance  -At risk for advancing scoliosis* | -Consider gait study  -Intermittent PT to address changes in function, gait, ROM, and/or strength, and to promote healthy, active lifestyle; often necessary to increase PT involvement to reduce risk of decline in functional mobility  -Trunk strengthening/stability  -Orthotics (AFOs, SMOs, UCBs, FOs) to maintain proper foot posture  -Regular strength training  -Continue regular exercise through community with adaptive sports and exercise classes  -Focus on ROM, specifically ankle dorsiflexion (with neutral midfoot), knee extension and hip extension with prolonged stretching  -Balance activities  -Referral as needed to orthopedic provider for spine, hip, and foot/ankle management  -Consider aquatherapy, hippotherapy, and strengthening based therapies  -Avoid use of botulinum toxin (“botox”) |
| Adult | -Monitoring for decline in gait and endurance  -Decreased ROM is not necessarily spasticity  -Trunk hypotonia  -Scoliosis* | -Consider gait study  -Intermittent PT to address changes in function, gait, ROM, and/or strength, and to promote healthy, active lifestyle  -Continued need for monitoring ROM, specifically ankle dorsiflexion (with neutral midfoot), knee extension and hip extension  -Orthotics (AFOs, SMOs, UCBs, FOs) to maintain proper foot posture  -Regular strength training  -Continue regular exercise through community with adaptive sports and exercise classes  -Consider aquatherapy, hippotherapy, and other strengthening based therapies  -Referral as needed to orthopedic provider for spine, hip, and foot/ankle management  -Avoid use of botulinum toxin (“botox”) |

 *monitored by orthopedic provider and interventions may include thoraco-lumbar jackets/orthoses, surgical procedures, increasing mobility, and facilitation of goof postures that engage muscles of the trunk
