# Supplementary material for: A multidisciplinary approach and consensus statement to establish standards of care for Angelman syndrome
Source: Mol Genet Genomic Med. 2022 Feb 11;10(3):e1843. doi: 10.1002/mgg3.1843 (PMC8922964; doi:10.1002/mgg3.1843)
Supplement: Supplementary file 5 — Table S4 [file MGG3-10-e1843-s001.docx]

**Supplemental Table 4: Occupational Therapy Interventions for Common Characteristics Present Throughout Life Span**

| **Characteristic** | **Symptoms** | **Interventions** |
| --- | --- | --- |
| Sensory Characteristic: Distress during grooming tasks such as toileting, teeth brushing, nail trimming^1^ | Elopement, aggression, resistance toward hand over hand assistance, increased distress during task | Calm, soothing environment and predictable routine  Pair grooming tasks with highly preferred activity (crinkly textures, videos, looking at self in mirror, water play) to serve as distraction  Offer hand under hand assistance to make task easier to perform  Teach child to perform steps of task by using forward and backward chaining  Change sensory properties of tools (electric toothbrush vs regular toothbrush)  Reward cooperative behaviors during grooming activities  Procedural reminder strips to outline steps in the grooming sequence  Video modeling |
| Sensory Characteristic: Mouthing of Objects^1^ | Licking, sucking, chewing, swallowing of non-food items; increased hand-to- mouth behavior | In toddlerhood and childhood - select toys which are less likely to be sought out for mouthing:   - Avoid soft rubber toys or other materials preferred by person to mouth - Activities or toys which have a variety of textures but are attached and not able to be brought to the mouth (book with tactile properties, cause and effect toys)   Ensure the mouth is occupied with a replacement (chewy, straw, oral motor tool) during fine motor tasks  Encourage manipulation of objects with hands instead of putting in mouth   - Only allow hand manipulation of targeted toys when teaching new skills and remove briefly if attempted to place in mouth - Short repetition of tasks to hold and capture attention as sometimes mouthing appeared to occur with loss of interest in task   Teach appropriate replacement behaviors   - hand licking to wiping on napkin/towel   Provide Sensory exploration in down time with   - Developmentally appropriate mouthing toys starting in infancy - Ensuring foods provided during meals times are crunchy, sour, spicy, chewy foods; must be aware of any dietary restrictions and cleared by speech-language pathologist for safe eating |
| Sensory Characteristic: Hand Flapping^1^ | Bilateral, unilateral movement of arms and or hands | In most cases intervention not recommended but if important patient or family or impeding function:   - May be shaped into clapping or waving - May be redirected into heavy work tasks during routines |
| Sensory Characteristic: Rough Housing/Aggressive Behavior^1^ | Grabbing people, knocking things over, dumping things out, pinching, hair pulling | Any changes in behavior should be explored as a possible expression of discomfort, pain or illness  Teach appropriate play and social interaction skills  found that teaching functional communication to replace behaviors such as grabbing, pinching reduced their frequency in children with Angelman Syndrome. OTs play vital role in collaboration with SLP and behavioral therapists:   - Collaborate with behavioral and SLP to determine function of and possible replacements for behavior - Assist with increasing adaptive access to communication - Recommend accessible gesture strategies to fulfill function   Movement during learning activities (child can walk across room to retrieve physical items related to literacy or recognition instead of remaining seated at table) (Sheldon, 2017)  Provide frequent opportunities for heavy work  Alternate between calming and active activities to help with regulation |
| Sensory Characteristic: hyporesponsive to tactile/ vestibular input^1^ | Seeking intensive movement and wanting to touch, mouth undesirable items | ^2^ |
| Self-Regulation: Excitability/ Decreased Attention Span | Hyperactivity, overstimulation, decreased ability to remain seated, dysregulation, highly excitable, frequent laughter | Intentional Use of Co-Regulation   - make interactions with individuals positive - eye contact and smiling are particularly motivating - refrain from overly excitable greetings that can be dysregulating, antecedent to negative behaviors   Integrate heavy work into daily routines and activities  Modify environment to reduce sensory overload:   - decrease visual distractions - seat the person facing the door with nothing happening behind him/her - when distracted, verbalize what the person is seeing/hearing and refer back to the task - consider adaptive seating to provide input   throughout the day  Increased opportunities for movement, provide deep pressure to increase focus and calm  Reward on task behavior   - Social rewards like smiles, high fives, praise, preferred play activities most motivating while monitoring for overstimulation and excitement |
| Visual Motor: Access to AAC strategies and devices | Fine motor deficits, upper extremity tremor, lack of isolated index finger; cortical visual impairment (CVI) | Positioning of device for best access visually and motor accuracy  Consult with SLP to assess the impact of balance deficits on independent transportability of device  Consult with SLP to match motor skill ability to most accessible strategy/device   - Help to adapt and shape gestures the person can imitate - A device must be a good match for the child’s abilities in order for child to accept it^3^ - For a dynamic device OT may consult with SLP or AAC evaluation team about current fine motor skills impacting : - button sizing, spacing between buttons colors or background of buttons, need for a key guard, number of exposed buttons at a time   Recommend motivating toys or activities and setting up the environment to sustain attention which can be utilized to inspire, motivate and empower purposeful communication  Use matte finishing when laminating printed AAC materials |
| Motor Skills affecting safe navigation of home and community, development of fine motor or self-help skills | Movements vary from minor to more severe jerky movements^4^  low tone, mixed muscle, or high muscle tone depending on the individual^5^ | Least amount of tactile assistance for sitting or standing balance required is recommended with fading of support  Attempt reaching and fine motor tasks in standing to improve control  Shoulder girdle and core strengthening activities  Toy or activity modification  Toy Selection and adaptation as needed based on motor, visual and sensory needs of children   - Consider use of switch toys if access to cause and effect toys are limited due to motor limitations   Gastroesophageal reflux impacting positioning tolerance in infancy:   - Doctor may have provided orders to parents to avoid positions to prevent pain and discomfort which will inform treatment and positioning recommendations t |
| Activities of Daily Living (ADL): Toileting | Decreased access and independence to toileting | Modify environment: toilet seating, stools, grab bars, optimize positioning to improve access and safety  Collaborate with behavioral and speech therapists to create consistent, structured routine opportunities and to allow for communication regarding toileting needs |
| ADL:  Feeding difficulties | Gastroesophageal reflux, tongue thrusting or uncoordinated tongue movements^4^  refuse new foods^1^  difficulty with utensil manipulation | Work with doctor and SLP to ensure safety with eating and to determine if swallow study is required  OT experienced and specialized in feeding recommended or work closely with SLP  Ensure proper positioning with feet planted and ample hip, back and lateral support  Environmental modifications including limited visual and auditory distractions during feeding  Take into account visual concerns and make modifications as necessary. For example, with a child with Cortical Visual Impairment ( CVI) use a food or spoon of their preferred color which contrasts with the tray or plate  Utensil Use:   - encourage adaptive handle, built up spoon - Provide one loaded spoonful or one loaded fork at a time - Weighted spoon to increase control - Use of non-skid placement or suction cupped plates or bowls - Allow messy exploration of food and textures with hands as soon as able, encourage hand to mouth behavior |
| ADL: Sleep | Difficulties and safety concerns with sleeping | Create a safe sleep environment, keep consistent sleep and wake times , and coach parents on how to respond during time expected to sleep^6^  Diminish or de-intensify parent-child interactions during the night.  Make sure the child is not dressed too warm as individuals with AS have bad heat tolerance.  Consider safety and prevent access to kitchen and bathroom while unsupervised during sleeping hours |
| Participation in meaningful leisure in adolescence and adulthood | Lack of access to appropriate leisure | Independence documented with swimming, adaptive biking, and yoga in adulthood in people with Angelman Syndrome^7^ |
| Participation in meaningful roles and routines as adult in adulthood |  | Teach these skills using a combination of visual schedules, backward chaining and video modelling  Provide opportunities for social integration, such as visits to malls, sports and recreational centers  Create structured daily jobs such a pushing in chairs at the table, helping to move laundry or boxes, getting the mail, taking out garbage at home  Educate community on how to socially interact and engage with individuals by explaining the use AAC strategies and devices |
