# Supplementary material for: A multidisciplinary approach and consensus statement to establish standards of care for Angelman syndrome
Source: Mol Genet Genomic Med. 2022 Feb 11;10(3):e1843. doi: 10.1002/mgg3.1843 (PMC8922964; doi:10.1002/mgg3.1843)
Supplement: Supplementary file 6 — Table S5 [file MGG3-10-e1843-s005.docx]

| Diet | Fat, % calories | Protein,  % calories | Carbohydrate (gm), % calories | Initiation/Considerations |
| --- | --- | --- | --- | --- |
| Regular diet | 30% | 20% | 50% |  |
| Classic KD 4:1 ratio | 90% | 6% | 4% | Usually inpatient; ideal for patients < 2 years of age |
| MAD | Unrestricted encouraged | Unrestricted | 10-20, 20% | Outpatient; older children and adolescents. Easier meal planning. |
| LGIT | 60% | 20-30% | 40-60, 10-20% | Outpatient; older children and adolescents. Low glycemic index<50 |
